# The Oxysterol Receptor EBI2 Links Innate and Adaptive Immunity to Limit IFN Response and Systemic Lupus Erythematosus

**DOI:** 10.1002/advs.202207108

**Published:** 2023-07-19

**Authors:** Fang Zhang, Baokai Zhang, Huihua Ding, Xiangyue Li, Xilin Wang, Xiaomin Zhang, Qiaojie Liu, Qiuyun Feng, Mingshun Han, Longlong Chen, Linlin Qi, Dan Yang, Xiaojing Li, Xingguo Zhu, Qi Zhao, Jiaqian Qiu, Zhengjiang Zhu, Huiru Tang, Nan Shen, Hongyan Wang, Bin Wei

**Affiliations:** ^1^ Institute of Geriatrics Affiliated Nantong Hospital of Shanghai University (The Sixth People's Hospital of Nantong) School of Medicine Shanghai University Nantong 226011 China; ^2^ Immune Cells and Human Diseases Lab, Shanghai Engineering Research Center of Organ Repair School of Life Sciences Shanghai University Shanghai 200444 China; ^3^ Cancer Center Shanghai Tenth People's Hospital School of Medicine Tongji University Shanghai 200072 China; ^4^ Shanghai Institute of Rheumatology Renji Hospital Shanghai Jiao Tong University School of Medicine (SJTUSM) Shanghai 200127 China; ^5^ State Key Laboratory of Virology Wuhan Institute of Virology Chinese Academy of Sciences University of Chinese Academy of Science Wuhan 430071 China; ^6^ State Key Laboratory of Cell Biology Shanghai Institute of Biochemistry and Cell Biology Center for Excellence in Molecular Cell Science Chinese Academy of Sciences University of Chinese Academy of Sciences Shanghai 200031 China; ^7^ State Key Laboratory of Genetic Engineering School of Life Sciences Human Phenome Institute Zhangjiang Fudan International Innovation Center Zhongshan Hospital Fudan University Shanghai 200032 China; ^8^ Metabonomics and Systems Biology Laboratory at Shanghai International Centre for Molecular Phenomics Fudan University Shanghai 200032 China; ^9^ Interdisciplinary Research Center on Biology and Chemistry Shanghai Institute of Organic Chemistry Chinese Academy of Sciences Shanghai 200032 China; ^10^ School of Life Science Hangzhou Institute for Advanced Study University of Chinese Academy of Sciences Hangzhou 310024 China; ^11^ Department of Laboratory Medicine Gene Diagnosis Research Center Fujian Key Laboratory of Laboratory Medicine The First Affiliated Hospital Fujian Medical University Fuzhou 350000 China

**Keywords:** Epstein–Barr virus‐induced gene 2, interferon, macrophages, oxysterols, systemic lupus erythematosus

## Abstract

Systemic lupus erythematosus (SLE) is a complex autoimmune disease with abnormal activation of the immune system. Recent attention is increasing about how aberrant lipid and cholesterol metabolism is linked together with type I interferon (IFN‐I) signaling in the regulation of the pathogenesis of SLE. Here, a metabonomic analysis is performed and increased plasma concentrations of oxysterols, especially 7*α*, 25‐dihydroxycholesterol (7*α*, 25‐OHC), are identified in SLE patients. The authors find that 7*α*, 25‐OHC binding to its receptor Epstein–Barr virus‐induced gene 2 (EBI2) in macrophages can suppress STAT activation and the production of IFN‐*β*, chemokines, and cytokines. Importantly, monocytes/macrophages from SLE patients and mice show significantly reduced EBI2 expression, which can be triggered by IFN‐*γ* produced in activated T cells. Previous findings suggest that EBI2 enhances immune cell migration. Opposite to this effect, the authors demonstrate that EBI2‐deficient macrophages produce higher levels of chemokines and cytokines, which recruits and activates myeloid cells,T and B lymphocytes to exacerbate tetramethylpentadecane‐induced SLE. Together, via sensing the oxysterol 7*α*, 25‐OHC, EBI2 in macrophages can modulate innate and adaptive immune responses, which may be used as a potential diagnostic marker and therapeutic target for SLE.

## Graphical abstract

This study has identified the increased plasma 7*α*, 25‐dihydroxycholesterol in patients with systemic lupus erythematosus (SLE), which binds to Epstein–Barr virus‐induced gene 2 (EBI2) to inhibit the type I interferon (IFN‐I) response in macrophages. IFN‐*γ* stimulation reduces EBI2  expression to induce increased levels of IFN‐I and chemokines, resulting in massive infiltration and activation of T/B lymphocytes to exacerbate SLE.

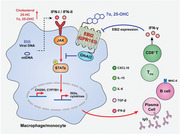

## 1 Introduction

Systemic lupus erythematosus (SLE) is a complex autoimmune disease with different pathogenic mechanisms, leading to extremely varied clinical manifestations, including kidney damage, arthritis, skin disease, blood cell abnormalities, and neurological complications.^[^ ^1^ ^]^ Alongside genetic factors, environmental triggers, and hormones,^[^ ^2^ ^]^ recent studies have highlighted the role of mitochondrial dysfunction, defective clearance of apoptotic cells, or inefficient degradation of DNA‐containing neutrophil extracellular traps (NETs) in the initiation of SLE.^[^ ^3^ ^]^ Notably, abnormal release of nuclear acids and cellular contents from damaged mitochondria, apoptotic cells, or NETs could be sensed by pattern recognition receptors (PRRs) to activate the innate immune system, leading to type I interferon (IFN‐I) production. A rise in serum IFN‐I levels has been noted in SLE patients,^[^ ^4^ ^]^ and IFN‐I is positively correlated with the SLE disease activity index (SLEDAI) scores and anti‐dsDNA (double‐stranded DNA) levels.^[^ ^1b^ ^]^ Moreover, many SLE patients display an increased expression of IFN‐stimulated genes (ISGs) (referred as IFN signature) in multiple types of immune cells.^[^ ^5^ ^]^ Critically, anifrolumab, a monoclonal antibody antagonist of the IFN‐I receptor (IFNAR), was recently approved in the United States for the treatment of adult patients with moderate to severe SLE who are receiving standard therapy.^[^ ^6^ ^]^ Hence, IFN‐I and IFN‐I‐induced responses have drawn increasing attention to understand SLE pathogenesis.

Macrophages are located in multiple organs or tissues, which can respond to diverse stimuli to polarize into different subsets, that is, producing pro‐inflammatory or anti‐inflammatory cytokines, and expressing different receptors to activate or inhibit T cells. This macrophage function plasticity could impact the development of SLE.^[^ ^7^ ^]^ Upon recognizing exogenous nuclear acids or endogenous inappropriately aggregated self‐DNA, PRRs such as toll‐like receptors (TLRs), cytosolic cGAS (cyclic guanosine monophosphate‐adenosine monophosphate [cGAMP] synthase), or RIG‐I‐like receptors were activated to trigger a series of signaling cascades for the activation of the transcription factors IFN‐regulatory factor 3 (IRF3) and nuclear factor‐*κ*B (NF‐*κ*B) to produce IFN‐I and pro‐inflammatory cytokines.^[^ ^8^ ^]^ IFN‐I then activates the IFNAR‐mediated signaling pathway, leading to the transcription of a wide range of ISGs, including multiple chemokines that further recruit numerous types of immune cells to amplify SLE. Accordingly, we aimed to investigate key molecules in macrophages that can precisely control IFN‐I and ISGs during the development of SLE, which might introduce potential targets for the diagnosis or treatment of SLE.

Mounting evidences have shown that dyslipidemia is prevalent in SLE patients.^[^ ^9^ ^]^ About 30% SLE patients exhibit hypercholesterolemia, which rises to over 60% 3 years after diagnosis.^[^ ^10^ ^]^ Sex hormones, derivatives of cholesterol, have been demonstrated to closely related to the development and susceptibility of SLE.^[^ ^11^ ^]^ Excitingly, several advanced studies have indicated that cholesterol or its intermediates could regulate IFN‐I response. Opposite to that reducing cholesterol biosynthesis flux could enhance IFN‐I and ISGs expression in macrophages,^[^ ^12^ ^]^ our previous study revealed that the accumulation of the cholesterol precursor 7‐dehydrocholesterol (7‐DHC) could promote IFN‐I production in macrophages against viral infection.^[^ ^13^ ^]^ Consequently, it is crucial to identify which cholesterol metabolites are abnormally produced and are functionally linked with IFN and ISGs to control the pathogenesis of SLE.

In this study, we conducted an assessment of the expression levels of enzymes and metabolites relevant to cholesterol synthesis, uptake, efflux, and esterification. The plasma concentration of 7*α*, 25‐dihydroxycholesterol (7*α*, 25‐OHC) was markedly increased along with the enhanced expression of the enzyme 25‐hydroxycholesterol 7alpha‐hydroxylase (CYP7B1) in peripheral blood mononuclear cells (PBMCs) of SLE patients. 7*α*, 25‐OHC was identified as the most potent endogenous ligand for the G protein‐coupled receptor (GPCR) Epstein–Barr virus‐induced gene 2 (EBI2, also known as GPR183).^[^ ^14^ ^]^ EBI2 and 7*α*, 25‐OHC have been elucidated to promote migration of immune cells to regulate many physiological and pathological processes. EBI2 mediates migration of T cells to the outer T zone to augment follicular helper T (T_FH_) cell differentiation,^[^ ^15^ ^]^ and promotes migration of encephalitogenic T_H_17 cells into the central nervous system to aggravate experimental autoimmune encephalomyelitis.^[^ ^16^ ^]^ Additionally, EBI2 directs B cells to the outer follicular and induces early plasmablast responses.^[^ ^17^ ^]^ Since abnormal production of IFN and activation of T/B cells are important for the development of SLE, we asked whether 7*α*, 25‐OHC/EBI2 affect the production of IFN and ISGs in innate immune cells, which then might promote the cross‐talk with adaptive immune cells to regulate the development of SLE.

In this study, we have identified the increased plasma 7*α*, 25‐OHC in SLE patients, which binds to EBI2 in macrophages to reduce the production of IFN‐I and ISGs. EBI2‐deficient macrophages augment the production of IFN‐I and chemokines, resulting in massive infiltration and activation of myeloid cells and T and B lymphocytes to exacerbate the development of SLE. We therefore propose a new role for EBI2 in macrophages, whereby it modulates innate and adaptive immune responses to protect the host from SLE.

## 2 Results

### 2.1 Abnormal Cholesterol and Oxysterol Metabolism in Blood from SLE Patients

**Figure 1:**
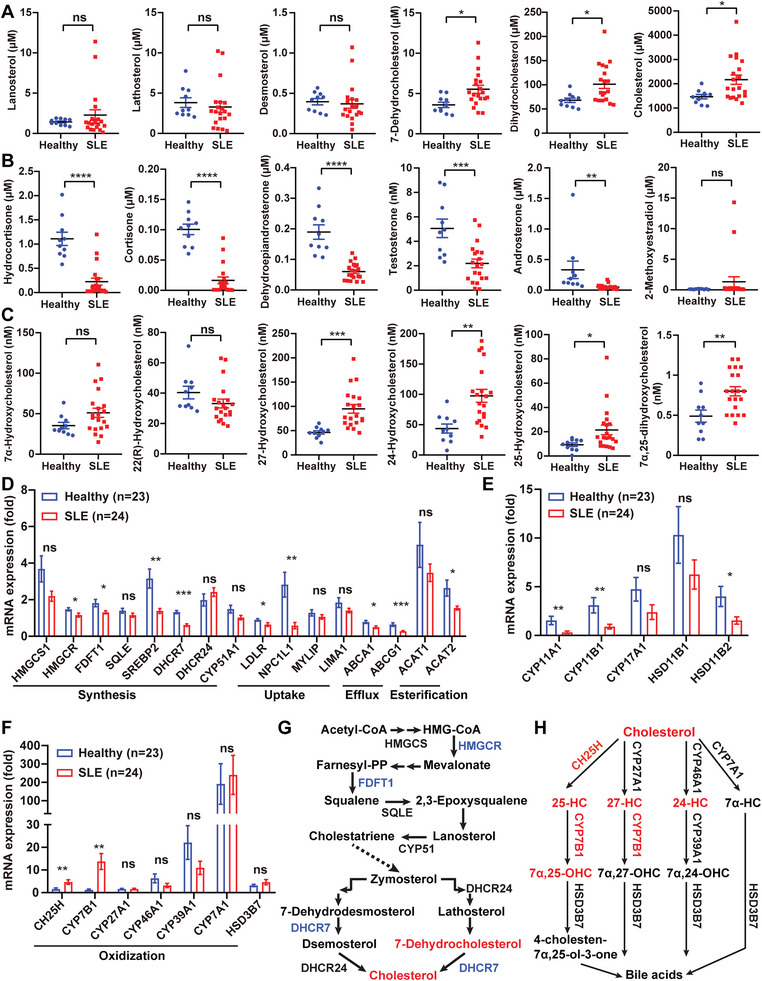
Abnormal cholesterol and oxysterol metabolism in blood from SLE patients. A–C) Plasma concentrations of cholesterol synthesis (A), oxidization (B), and metabolism (C) intermediates in healthy donors (*n* = 10) and SLE patients (*n* = 20) by LC‐MS. D–F) The relative mRNA expression of cholesterol synthesis, oxidization, and metabolism‐related enzyme genes in PBMCs of healthy donors (*n* = 23) and SLE patients (*n* = 24). G,H) Schematic diagrams of cholesterol (G) and oxysterols (H) biosynthesis pathways. The up‐regulated intermediates or enzyme genes were shown in red, while the down‐regulated intermediates or enzyme genes were shown in blue. Data are shown as mean ± SEM. ns, not significant (*p* > 0.05); *p < 0.05, **p < 0.01, ***p < 0.001, and ****p < 0.0001, using a two‐tailed, unpaired Student's *t*‐test (A–F).

Emerging evidence suggests that lipid and cholesterol metabolism has a complex impact on the pathogenesis and progression of SLE.^[^ ^18^ ^]^ To identify the dominant disrupted cholesterol metabolism in SLE patients, we used liquid chromatography‐mass spectrometry (LC‐MS) to detect cholesterol intermediates in the plasma of SLE patients. Plasma concentrations of cholesterol, 7‐DHC and dihydrocholesterol in SLE patients were higher than in healthy controls (HCs); furthermore, other cholesterol intermediates including lanosterol, lathosterol, and desmosterol were not altered (**Figure** **1**A). Conversely, the levels of steroid hormones, including glucocorticoids (hydrocortisone and cortisone) and androgens (dehydroepiandrosterone, testosterone, and androsterone) were reduced in the plasma of SLE patients, although estrogen such as 2‐methoxystradiol, estradiol benzoate, and pregnenolone did not differ (Figure 1B and Figure S1A, Supporting Information). Decreased serum/plasma levels of cortisol, dehydroepiandrosterone and testosterone have been previously reported in SLE patients.^[^ ^19^ ^]^ Importantly, we discovered that several oxysterols including 24‐hydroxycholesterol (24‐HC), 25‐HC, 27‐HC, and 7*α*, 25‐OHC were prominently increased in the plasma of SLE patients (Figure 1C, except for 7*α*‐HC and 22 (R)‐HC).

Considering that abnormal changes in cholesterol and its intermediates are mainly regulated by a series of metabolic enzymes, we examined the mRNA levels of enzymes involved in cholesterol synthesis, uptake, efflux, and esterification. In PBMCs of SLE patients, most of these metabolic enzymes either declined or remained unchanged (Figure 1D), which was inconsistent with the elevated levels of metabolites. To explain this inconsistency, we propose that when abnormal cholesterol metabolism occurs in SLE patients,^[^ ^20^ ^]^ the body may attempt to restore disrupted cholesterol balance toward homeostasis, possibly by altering the expression of certain metabolic enzymes. Additionally, the mRNA levels of some enzymes responsible for catalyzing cholesterol to steroid hormones were reduced (Figure 1E). In contrast, we observed significant up‐regulation of cholesterol 25‐hydroxylase (CH25H) and CYP7B1 (Figure 1F), which aligns with the enhanced levels of the metabolites named 25‐HC and 7*α*, 25‐OHC (Figure 1C). Flow diagrams illustrating cholesterol biosynthesis and the generation of different derivatives were presented in Figure 1G,H and Figure S1B, Supporting Information, with blue labels indicating reduced enzymes or metabolites and red labels representing enhanced enzymes or metabolites. Moreover, we observed similar changes in the levels of certain cholesterol metabolites and enzymes in peritoneal irrigation fluid (PIF) or peritoneal cells of tetramethylpentadecane (TMPD)‐induced SLE mice, particularly an increase in CH25H and 25‐HC for the generation of 7*α*, 25‐OHC (Figure S1C,D, Supporting Information). We further confirmed the increased concentration of 7*α*, 25‐OHC in the serum of TMPD‐induced mice (Figure S1E, Supporting Information). Together, our findings have demonstrated abnormal cholesterol metabolism in the blood of SLE patients, highlighting the up‐regulation of 7*α*, 25‐OHC as particularly intriguing.

### 2.2 The Down‐Regulated Oxysterol Receptor EBI2 in Monocytes of SLE Patients

**Figure 2:**
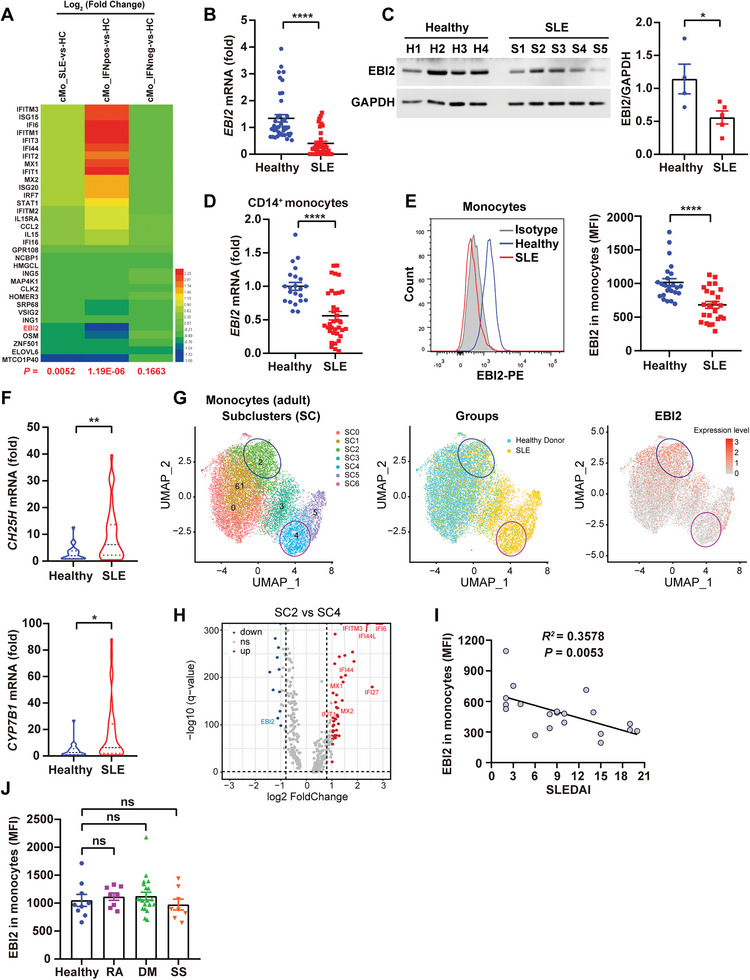
The down‐regulated oxysterol receptor EBI2 in monocyte of SLE patients. A) Heat map of the DEGs in cMos of SLE (IFN‐positive or IFN‐negative) patients versus HCs according to the GEO database (GSE149050). B) *EBI2* mRNA levels in PBMCs of healthy donors (*n* = 41) and SLE patients (*n* = 36). C) The protein expression of EBI2 in PBMCs of healthy donors (*n* = 4) and SLE patients (*n* = 5). D,F) The mRNA levels of *EBI2* (D) and *CH25H* and *CYP7B1* (F) in purified CD14^+^ monocytes of healthy donors (*n* = 22) and SLE patients (*n* = 35). E) Surface EBI2 expression on CD11b^+^CD14^+^ monocytes from PBMCs of healthy donors (*n* = 24) and SLE patients (*n* = 24). G) scRNA‐seq data of monocytes from adult SLE patients or healthy donors were classified into molecularly distinct subclusters (SCs). UMAP plots representing SCs (*n* = 6), groups (healthy donors or SLE), and the expression values of EBI2. H) Volcano plot of DEGs from SC2 (EBI2^hi^) and SC4 (EBI2^lo^). I) Correlation between EBI2 expression in monocytes and the disease activity was measured by the SLEDAI (*n* = 20). J) Surface EBI2 expression on monocytes of healthy donors (*n* = 9) and patients with RA*(n* = 8), DM(*n* = 21), or SS (*n* = 8). Data are shown as mean ± SEM. *p < 0.05, **p < 0.01, and ****p < 0.0001, using a two‐tailed, unpaired Student's *t*‐test (B–E [right panels], F), one‐way ANOVA with Holm–Sidak's multiple comparisons test (J), or Pearson's test (I).

Aberrant cholesterol metabolism may lead to changes in the activation of cellular signaling pathways, thereby affecting cellular functions. We therefore analyzed RNA sequencing (RNA‐seq) data in the Gene Expression Omnibus (GEO) database^[^ ^5b^ ^]^ to investigate cholesterol‐related differentially expressed genes (DEGs) in PBMCs of SLE patients. Undoubtedly, ISGs were significantly up‐regulated in classical monocytes (cMos) of SLE patients. Surprisingly, we identified the down‐regulated transcription of *EBI2*, the receptor for 7*α*, 25‐OHC, in cMos of SLE patients (*p* = 0.0052). Importantly, EBI2 expression was further decreased in cMos from IFN‐positive SLE patients (*p* < 0.0001), in contrast to normal expression in IFN‐negative SLE patients (*p* = 0.1663) (**Figure** **2**A). Again, based on multiplexed single‐cell RNA‐seq (scRNA‐seq) data from a different study,^[^ ^21^ ^]^ *EBI2* was among the top 11 down‐regulated genes in cMos of SLE patients with Asian ancestry (Figure S2A, Supporting Information). To verify this finding, we collected PBMCs and CD14^+^ monocytes from SLE patients and healthy donors to determine the transcription of *IFNA*/*B* and *EBI2*. In contrast to enhanced transcription of *IFNA*/*B* (Figure S2B, Supporting Information), mRNA and protein expression of EBI2 was significantly reduced in PBMCs of SLE patients (Figure 2B,C). Notably, the percentages of monocytes were substantially increased in PBMCs of SLE patients (Figure S2C, Supporting Information), and the decreased transcription and surface expression of EBI2 were confirmed in monocytes of SLE patients (Figure 2D,E). In both PBMCs and CD14^+^ monocytes of SLE patients, mRNA levels of the enzymes for 7*α*, 25‐OHC generation including CH25H and CYP7B1 were augmented (Figures 1F and 2F). We then analyzed the correlation between EBI2 and IFN using scRNA‐seq data from PBMCs of SLE patients published previously.^[^ ^22^ ^]^ Pediatric and adult SLE patients’ monocytes were clustered, followed by a 2D uniform manifold approximation and projection (UMAP), and then the EBI2‐high expression (EBI2^hi^) subset was compared with the EBI2‐low expression (EBI2^lo^) subset (Figure 2G and Figure S2D, Supporting Information). The volcano plot of DEGs revealed that the expression of EBI2 was negatively related to that of ISGs, such as *MX1*, *MX2*, and *IFI44L* (Figure 2H and Figure S2E, Supporting Information). Moreover, SLEDAI was inversely correlated with EBI2 expression in monocytes (Figure 2I). In addition, we found that EBI2 expression remained unchanged in the monocytes of patients with rheumatoid arthritis (RA), dermatomyositis (DM), and Sjögren's syndrome (SS) (Figure 2J). Together, we propose EBI2 as an IFN‐related gene, and its expression is reduced in monocyte from SLE patients.

### 2.3 Macrophages from SLE Patients and Mice Reduce EBI2 Expression by IFN‐*γ*

**Figure 3:**
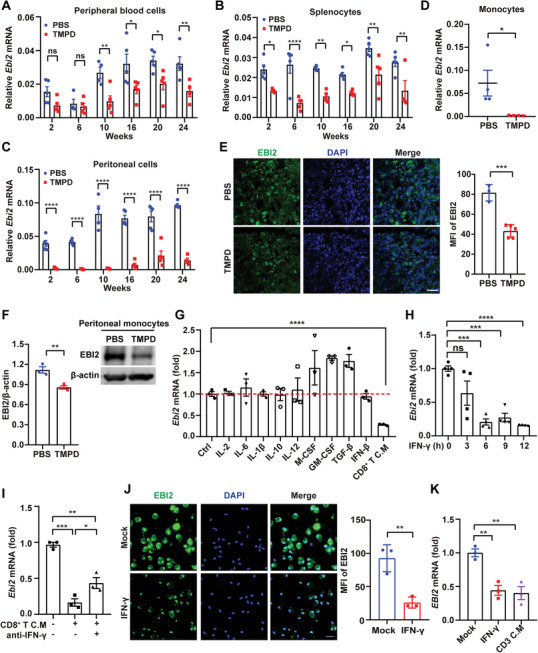
Macrophages from SLE patients and mice reduce EBI2 expression by IFN‐*γ*. A–C) *Ebi2* mRNA expression in peripheral blood cells (A), splenocytes (B), or peritoneal cells (C) of mice treated with PBS (*n* = 5, and *n* = 4 only at week 10) or TMPD (*n* = 5, and *n* = 4 only at week 24). D) *Ebi2* mRNA in splenic monocytes of PBS control (*n* = 4) or TMPD‐treated mice (*n* = 4). E) Immunofluorescence analysis of EBI2 expression in spleen of PBS control (*n* = 3) or TMPD‐treated mice (*n* = 5) (scale bar: 25 µm). F) EBI2 protein expression in peritoneal monocytes of mice after PBS (*n* = 3) or TMPD (*n* = 3) treatment for 12 weeks. G) *Ebi2* mRNA expression in PEMs after stimulated with various cytokines including IL‐2 (100 U mL^−1^), IL‐6 (40 ng mL^−1^), IL‐1*β* (10 ng mL^−1^), IL‐10 (10 ng mL^−1^), IL‐12 (10 ng mL^−1^), M‐CSF (20 ng mL^−1^), GM‐CSF (20 ng mL^−1^), TGF‐*β* (5 ng mL^−1^), IFN‐*β* (500 ng mL^−1^), or CD8^+^ T C.M for 24 h (*n* = 3). H) *Ebi2* mRNA expression in murine PEMs after stimulated with IFN‐*γ* (200 ng mL^−1^) for different time (*n* = 4). I) CD8^+^ T C.M was incubated with anti‐IFN‐*γ* antibody (10 µg mL^−1^) at 4 °C overnight, and then treated PEMs for 12 h to detect *Ebi2* mRNA expression (*n* = 3). J) Immunofluorescence analysis of EBI2 expression in PEMs after stimulation with IFN‐*γ* (200 ng mL^−1^) for 12 h (*n* = 3; scale bar: 20 µm). K) Human CD14^+^ monocytes were stimulated with IFN‐*γ* (200 ng mL^−1^) or CD3 C.M for 12 h to detect *EBI2* transcription (*n* = 3). Data are shown as mean ± SEM or representative from at least three independent experiments (E,J [left panels], F [right panel]). *p < 0.05, **p < 0.01, ***p < 0.001, and ****p < 0.0001, using one‐way ANOVA with Holm–Sidak's multiple comparisons test (G–I,K), two‐way ANOVA with Holm–Sidak's multiple comparisons test (A–C), or a two‐tailed, unpaired Student's *t*‐test (D–F,J).

We next investigated EBI2 expression in TMPD‐induced SLE mice, since this model exhibits many clinical features of lupus patients.^[^ ^23^ ^]^ The transcription of *Ebi2* was decreased in peripheral blood cells (**Figure** **3**A), splenocytes (Figure 3B), and most significantly in peritoneal cells (Figure 3C) of TMPD‐induced SLE mice, but showed minor or no changes in liver, kidney, lung, and heart (Figure S2F–I, Supporting Information). Consistently, monocytes sorted from spleens of TMPD‐induced SLE mice exhibited reduced *Ebi2* transcription (Figure 3D). We also verified the reduced protein expression of EBI2 in spleen (Figure 3E) and peritoneal monocytes (Figure 3F) of TMPD‐induced mice.

To explore how EBI2 was down‐regulated in monocytes/macrophages of SLE patients and mice, we first treated murine peritoneal exudate macrophages (PEMs) with 7*α*, 25‐OHC, which did not affect the mRNA and protein expression of EBI2 (Figure S2J,K, Supporting Information). As cytokines secreted by activated immune cells such as macrophages and T cells could play important roles in the development of SLE, we treated PEMs with various cytokines and supernatants from anti‐CD3/anti‐CD28‐activated CD8^+^ T cells (CD8^+^ T C.M) to screen which factors could reduce EBI2 expression. Intriguingly, we observed that activated CD8^+^ T cell supernatants significantly decreased *Ebi2* transcription in macrophages (Figure 3G). Furthermore, treatment with IFN‐*γ*, a crucial cytokine in CD8^+^ T C.M, substantially inhibited *Ebi2* transcription (Figure 3H), and neutralizing CD8^+^ T C.M with anti‐IFN‐*γ* antibody could partially restore *Ebi2* reduction (Figure 3I). A remarkable reduction in protein expression of EBI2 was also observed in IFN‐*γ*‐stimulated PEMs (Figure 3J). In agreement with this, *EBI2* transcription was down‐regulated in human CD14^+^ monocytes after treatment with supernatants from anti‐CD3/anti‐CD28‐activated human CD3^+^ T cells (CD3^+^ T C.M) or stimulation with IFN‐*γ* (Figure 3K). Taken together, these results suggest that cytokines produced by activated T cells including IFN‐*γ* could reduce EBI2 expression in monocyte/macrophages.

### 2.4 7*α*, 25‐OHC Inhibits EBI2 and GNAI3‐Mediated STAT Activation to Reduce IFN‐I Response Independent of TBK1

**Figure 4:**
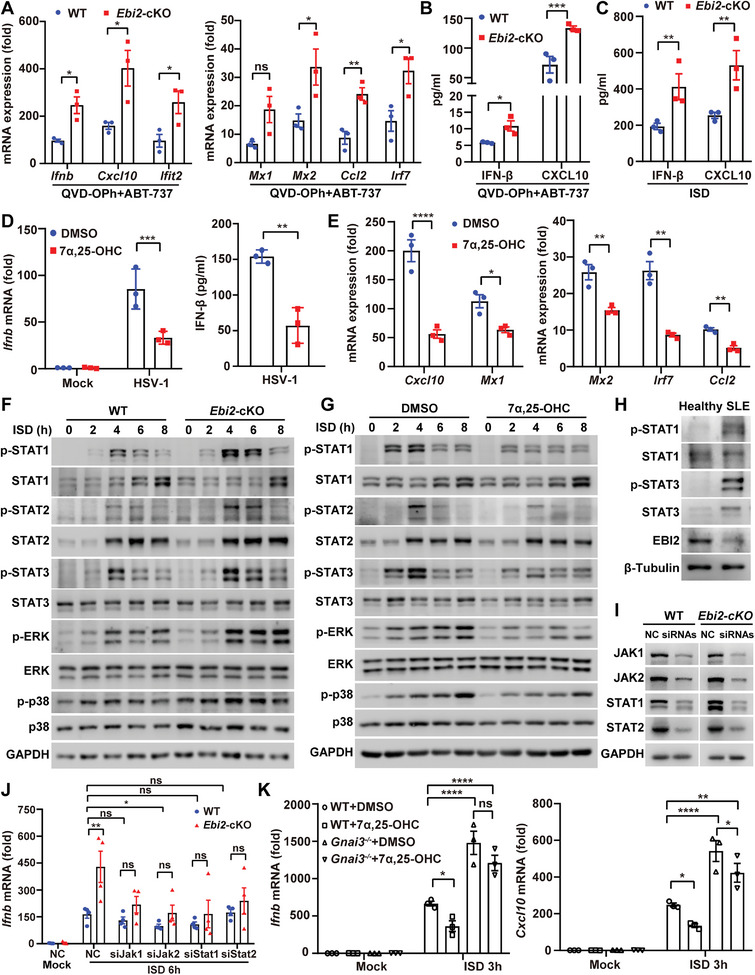
7*α*, 25‐OHC inhibits EBI2 and GNAI3‐mediated STAT activation to reduce IFN‐I response independent of TBK1. A,B) WT and *Ebi2*‐cKO PEMs were treated with QVD‐OPh (50 µm) plus ABT‐737 (5 µm) for 24 h to detect the mRNA or protein expression of IFN‐*β*, CXCL10 or *Ifit2*, *Mx1*, *Mx2*, *Ccl2*, and *Irf7* (*n* = 3). C) WT and *Ebi2*‐cKO PEMs were treated with ISD (5 µg mL^−1^) to detect the protein expression of IFN‐*β* and CXCL10 (*n* = 3). D,E) PEMs were pre‐treated with 7*α*, 25‐OHC (10 µM) for 2 h followed by HSV‐1 (multiplicity of infection (MOI), 1) infection for 6 h to detect IFN‐*β* production (D, *n* = 3), or infection for 12 h to detect the mRNAs of *Cxcl10*, *Mx1*, *Mx2*, *Irf7*, and *Ccl2* (E, *n* = 3). F) WT and *Ebi2*‐cKO PEMs were treated with ISD for different time to detect phosphorylation levels of STAT1, STAT2, STAT3, ERK, and p38. G) PEMs were pre‐treated with DMSO or 7*α*, 25‐OHC for 2 h followed by ISD stimulation for different time to detect the phosphorylation levels of STAT1, STAT2, STAT3, ERK, and p38. H) The phosphorylation levels of STAT1 and STAT3 in PBMCs of SLE patients and HCs. I,J) PEMs were transfected with siRNAs (40 nm) targeting *Jak1*, *Jak2*, *Stat1*, and *Stat2* for 48 h to detect the knockdown efficiency (I), or followed by ISD stimulation to measure the transcriptions of *Ifnb* (J, *n* = 4). K) WT and *Gnai3* ^−/−^ BMDMs were treated with DMSO or 7*α*, 25‐OHC for 2 h followed by ISD stimulation to measure the transcription of *Ifnb* and *Cxcl10* (*n* = 3). Data are shown as mean ± SEM or representative from three independent experiments (F–I). *p < 0.05, **p < 0.01, ***p < 0.001, and ****p < 0.0001, using two‐way ANOVA with Holm–Sidak's multiple comparisons test (D [left panel], J,K), or a two‐tailed, unpaired Student's *t*‐test (A–C,E).

Next, we asked whether EBI2 could regulate IFN‐I or ISG production during the progression of SLE. To this end, we generated *Ebi2* conditional knockout (cKO) mice bearing two loxP sites flanking the second exon of the *Ebi2* gene that were crossbred with the LysM‐Cre mice to specifically delete EBI2 in myeloid cells including macrophages (termed *Ebi2*‐cKO mice) (Figure S3A,B, Supporting Information). The genetic ablation efficiency of *Ebi2* in primary PEMs was confirmed by genome sequencing (Figure S3C, Supporting Information), quantitative PCR, and immunoblotting (Figure S3D, Supporting Information). *Ebi2*‐cKO mice have normal development of bone marrow‐derived macrophages (BMDMs), showing comparable percentages of macrophages (Figure S3E,F, Supporting Information) and normal expression of *Adgre1* and *Mertk* (Figure S3G, Supporting Information). It was reported that mitochondrial DNA (mtDNA) in the cytosol or extracellular milieu can drive IFN‐I production to promote human lupus.^[^ ^24^ ^]^ Therefore, we treated WT and *Ebi2*‐cKO PEMs with the BCL‐X_L_ and BCL‐2 inhibitor ABT‐737 plus the pan‐caspase inhibitor QVD‐OPh^[^ ^25^ ^]^ to release mtDNA into the cytoplasm, which increased the transcription of *Ifnb*, and ISGs including the chemokines *Cxcl10* and *Ccl2*, the GTP‐binding protein *Mx2*, the IFN‐induced protein with tetratricopeptide repeats 2 (*Ifit2*), *Irf7*, *Ifna4*, and *Ifna* in *Ebi2*‐cKO PEMs (**Figure** **4**A and Figure S4A, Supporting Information). The enhanced secretion of IFN‐*β* and CXCL10 in the culture supernatants of EBI2‐deficient macrophages was also confirmed (Figure 4B). Similar results were observed in ISD, a synthetic mimic of dsDNA, treated *Ebi2*‐cKO macrophages (Figure 4C and Figure S4B, Supporting Information). Upon poly (dA:dT) stimulation, a synthetic analog of B‐DNA, EBI2‐deficient macrophages also up‐regulated the production of *Ifnb* and *Cxcl10* (Figure S4C, Supporting Information). Because virus infection such as Epstein–Barr virus has been reported in SLE patients,^[^ ^26^ ^]^ we next infected EBI2‐deficient macrophages with the DNA virus herpes simplex virus 1(HSV‐1), which again resulted in a dramatic up‐regulation of *Ifnb* and *Cxcl10* (Figure S4D, Supporting Information).

We also investigated the effect of EBI2 on IFN‐I production in macrophages upon activation of TLR7/9, RIG‐I, or in response to RNA virus infection. To this end, we treated WT or *Ebi2*‐cKO PEMs with the TLR7 and TLR8 agonist resiquimod (R848), the TLR7 agonist imiquimod (R837), the TLR9 agonist CpG, the TLR3 and RIG‐I agonist poly (I:C), or the RNA virus vesicular stomatitis virus (VSV). *Ebi2*‐cKO PEMs showed increased transcription of *Ifnb* after CpG stimulation, lipofectamine 2000‐mediated transfection of poly (I:C), or VSV infection (Figure S4E, Supporting Information), and the enhanced secretion of IFN‐*β* was confirmed in poly (I:C)‐transfected *Ebi2*‐cKO PEMs (Figure S4F, Supporting Information). To further ascertain this phenotype, we constructed immortalized BMDMs (iBMDMs) that stably overexpressed human EBI2 (hEBI2) (Figure S4G, Supporting Information). EBI2‐overexpressed macrophages showed a remarkable down‐regulation of the mRNA levels of *Ifnb* and ISGs including *Cxcl10*, *Mx2*, *Irf7*, *Ifit2*, *Mx1*, and *Ccl2* (Figure S4H, Supporting Information) as well as IFN‐*β* secretion (Figure S4I, Supporting Information) in response to ISD stimulation.

Given that the ligand 7*α*, 25‐OHC was found to be enhanced in SLE patients, we investigated how it affected IFN‐I and ISG production. Addition of 7*α*, 25‐OHC substantially decreased the production of IFN‐*β* and ISGs, including *Cxcl10*, *Mx1*, *Mx2*, *Ccl2*, and *Irf7* after HSV‐1 infection (Figure 4D,E). Upon ISD and cGAMP stimulation, 7*α*, 25‐OHC treatment also reduced *Ifnb* transcription (Figure S4J,K, Supporting Information). These observations have elucidated that when macrophages sense stimuli such as cytosolic nucleic acids or viral infection, the binding of 7*α*, 25‐OHC to EBI2 could suppress the production of IFN‐I and ISGs.

To investigate EBI2‐related downstream signaling, we assessed the phosphorylation of TANK (TRAF family member associated NF‐*κ*B activator)‐binding kinase 1 (TBK1) and IRF3 in ISD‐stimulated *Ebi2*‐cKO macrophages. Surprisingly, we found that WT and *Ebi2*‐cKO PEMs showed comparable levels of phosphorylation in TBK1 and IRF3 (Figure S5A, Supporting Information). Since EBI2 inhibited the expression of ISGs, we next investigated its effect on the IFNAR signaling pathway. Indeed, ISD‐stimulated *Ebi2*‐cKO PEMs showed enhanced phosphorylation levels of STAT1, STAT2, STAT3, ERK, and p38 (Figure 4F and Figure S5B, Supporting Information). In agreement with this, 7*α*, 25‐OHC‐treated WT PEMs showed decreased phosphorylation of STAT1, STAT2, STAT3, ERK, and p38, but not TBK1 and IRF3 (Figure 4G and Figure S5C,D, Supporting Information).

Since we observed the elevated phosphorylation of STAT3 and STAT1 in PBMCs of SLE patients (Figure 4H and Figure S5E, Supporting Information), we asked whether blocking the JAK‐STAT signaling could reverse the enhanced IFN‐I response in EBI2‐deficient macrophages. Treatment with the pan‐JAK inhibitor tofacitinib or the JAK1 and TYK2 dual inhibitor PF‐06700841, as well as the STAT inhibitors SH‐4‐54 and static, could all abrogate the elevated transcription of *Ifnb* and *Cxcl10* in *Ebi2*‐cKO PEMs (Figure S6A, Supporting Information). We further confirmed that silencing *Jak1*, *Jak2*, *Stat1*, and *Stat2* could effectively restore the heightened expression of *Ifnb* and *Cxcl10* in ISD‐stimulated *Ebi2*‐cKO PEMs (Figure 4I,J and Figures S5F and S6B, Supporting Information). To verify the inhibitory role of EBI2 on IFN‐I production, we overexpressed hEBI2 in primary *Ebi2*‐cKO BMDMs using retrovirus infection. The infection efficiency of BMDMs was determined by detecting the percentages of GFP^+^ cells (Figure S6C, Supporting Information) or measuring the mRNA levels of *EBI2* by quantitative PCR (Figure S6D, Supporting Information). *Ebi2*‐cKO BMDMs enhanced the levels of *Ifnb*, *Cxcl10*, and IFN‐*β* secretion; but once EBI2 was overexpressed in *Ebi2*‐cKO BMDMs, the levels of *Ifnb*, *Cxcl10*, and IFN‐*β* were rescued and reduced to the levels comparable to those in WT BMDMs (Figure S6E,F, Supporting Information). In addition, the enhanced phosphorylation of STAT1 in *Ebi2*‐cKO BMDMs was abolished once EBI2 was overexpressed. In contrast, the phosphorylation of TBK1 and IRF3 was not affected by EBI2 deficiency or overexpression (Figure S6G,H, Supporting Information), which was consistent with our previous findings. These results suggest that 7*α*, 25‐OHC‐EBI2 signaling inhibits IFN‐I response through suppressing STAT activation.

Previous study has reported that EBI2 signals through the G protein alpha inhibitory family (G*α*_i_),^[^ ^27^ ^]^ which includes three subunits: G*α*_i1_, G*α*_i2_, and G*α*_i3_.^[^ ^28^ ^]^ Using small interfering RNAs (siRNAs) to silence *Gnai1*, *Gnai2*, *Gnai3*, and *Gnas*, respectively (Figure S7A, Supporting Information), we found that knockdown of *Gnai1*, *Gnai2*, or *Gnas* had no effect on the reduced expression of *Ifnb* by 7*α*, 25‐OHC treatment. However, knockdown of *Gnai3* abolished the 7*α*, 25‐OHC‐induced reduction of *Ifnb* transcription (Figure S7B, Supporting Information). Consistent with the effect by 7*α*, 25‐OHC, *Gnai3* did not affect phosphorylation of TBK1 and IRF3 (Figure S7D,E, Supporting Information); Instead, the attenuated phosphorylation of STAT1 and STAT3 (Figure S7F,G, Supporting Information), as well as the decreased expression of *Ifnb* and *Cxcl10* (Figure 4K), by 7*α*, 25‐OHC treatment were partially restored by *Gnai3* KO (Figure S7C, Supporting Information). This incomplete recovery of *Gnai3* ^−/−^ macrophages might be due to other receptors, such as retinoic acid‐related orphan receptor alpha, which can also recognize 7*α*, 25‐OHC.^[^ ^29^ ^]^ Importantly, we found that GNAI3 interacted with the tyrosine kinases JAK1, JAK2, and TYK2 and weakly bound to JAK3 (Figure S7H, Supporting Information). Together, we have elucidated that the 7*α*, 25‐OHC‐EBI2 axis inhibits STAT activation and IFN‐I response via the downstream GNAI3.

### 2.5 EBI2 Inhibits STAT Activation Depending on IFN‐I and IFN‐II Receptor

**Figure 5:**
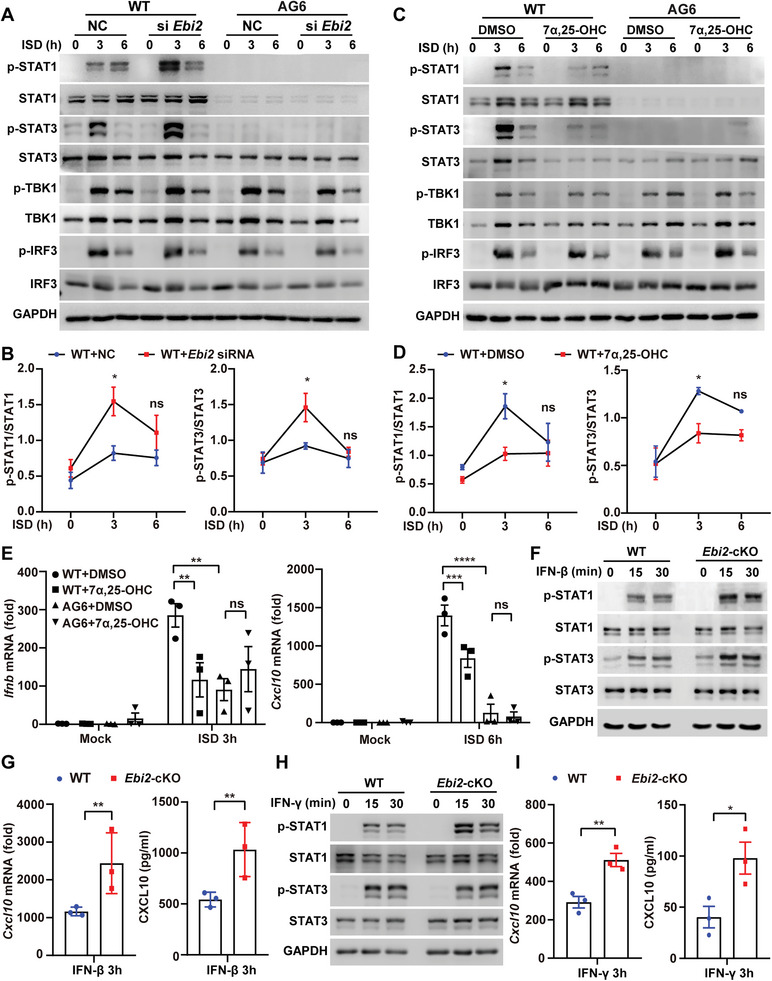
EBI2 inhibits STAT activation depending on IFN‐I and IFN‐II receptor. A,B) WT and AG6 PEMs were transfected with *Ebi2* siRNA for 48 h followed by ISD stimulation for 3 or 6 h to detect the levels of p‐STAT1, STAT1, p‐STAT3, STAT3, p‐TBK1, TBK1, p‐IRF3, and IRF3 (A) and quantify the p‐STAT1 and p‐STAT3 (B, *n* = 3). C–E) WT and AG6 PEMs were treated with DMSO or 7*α*, 25‐OHC for 2 h followed by ISD stimulation for 3 or 6 h to detect the levels of p‐STAT1, STAT1, p‐STAT3, STAT3, p‐TBK1, TBK1, p‐IRF3, and IRF3 (C) and quantify the p‐STAT1, p‐STAT3 (D, *n* = 3), or to detect the transcriptions of *Ifnb* and *Cxcl10* (E, *n* = 3). F–I) WT and *Ebi2*‐cKO PEMs were stimulated with IFN‐*β* (500 ng mL^−1^) or IFN‐*γ* (200 ng mL^−1^) for 15 or 30 min to detect the levels of p‐STAT1, STAT1, p‐STAT3, and STAT3 (F,H), or the expression of CXCL10 (G,I, *n* = 3). Data are shown as mean ± SEM or representative from three independent experiments (A,C,F,H). *p < 0.05, **p < 0.01, ***p < 0.001, and ****p < 0.0001, using two‐way ANOVA with Holm–Sidak's multiple comparisons test (B,D,E), or a two‐tailed, unpaired Student's *t*‐test (G,I).

Because IFN receptor‐mediated signaling could activate JAK/STAT, we next asked whether EBI2 was dependent on IFN receptors to inhibit STAT activation. PEMs from AG6 (IFN‐I and IFN‐II receptor deficient mice) and WT mice were transfected with *Ebi2* siRNA or treated with 7*α*, 25‐OHC, followed by ISD stimulation. We found that *Ebi2* knockdown or 7*α*, 25‐OHC treatment could enhance or reduce the phosphorylation levels of STAT1 and STAT3, respectively, in WT PEMs, whereas these effects were not observed in AG6 PEMs (**Figure** **5**A–D and Figure S8A,B, Supporting Information). Furthermore, in ISD‐stimulated AG6 macrophages, the 7*α*, 25‐OHC‐induced reduction of *Ifnb* and *Cxcl10* transcription was abrogated (Figure 5E). Consistently, in response to IFN‐*β* or IFN‐*γ* stimulation, the phosphorylation levels of STAT1 and STAT3 (Figure 5F and Figure S8C, Supporting Information) or STAT1 alone (Figure 5H and Figure S8D, Supporting Information), as well as the production of CXCL10 (Figure 5G,I) were enhanced in *Ebi2*‐cKO PEMs. These data suggest that EBI2 inhibits the effects of IFN‐I (i.e., STAT activation and ISG production), which is dependent on the IFN‐I/IFN‐II Receptor.

### 2.6 *Ebi2*‐cKO Lupus Mice Unexpectedly Increase Infiltration of Myeloid and T Cells

**Figure 6:**
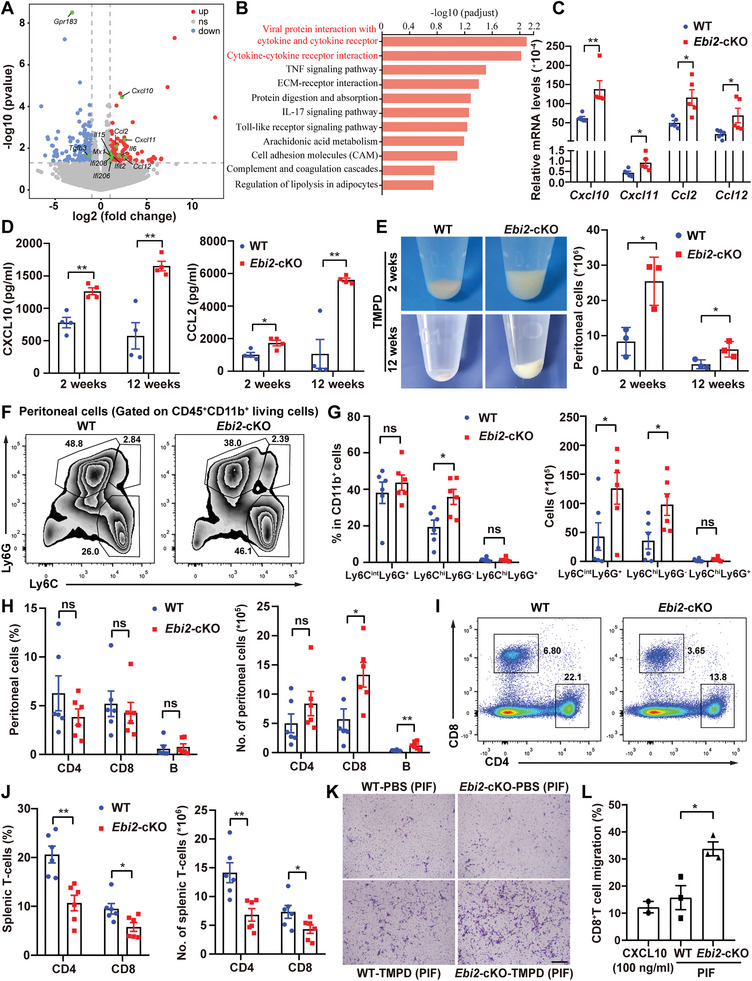
*Ebi2*‐cKO lupus mice unexpectedly increase infiltration of myeloid and T cells. A–C) RNA‐sequencing analysis of the DEGs (A), or KEGG pathway enrichment analysis of the DEGs (B), or mRNA expression of *Cxcl10*, *Cxcl11*, *Ccl2*, and *Ccl12* (C, *n* = 5) in the sorted monocytes from peritoneal cells of WT and *Ebi2*‐cKO mice with TMPD treatment for 2 weeks. D,E) WT and *Ebi2*‐cKO mice were intraperitoneally injected with TMPD for 2 or 12 weeks to detect the secretion of CXCL10 and CCL2 in the PIF (D, *n* = 4), or the cell numbers in the peritoneal cavity (E, *n* = 3). F–J) WT and *Ebi2*‐cKO mice were intraperitoneally injected with TMPD for 12 weeks to detect the percentages and numbers of Ly6C^int^Ly6G^+^ (neutrophils), Ly6C^hi^Ly6G^−^ (monocytes), Ly6C^hi^Ly6G^+^ cells (F,G, *n* = 6), T and B cells in peritoneal cavity (H, *n* = 6), or the percentages and numbers of T cells in spleens (I,J, *n* = 6). K,L) Transwell assay to check migration ability of murine PEMs (K) or anti‐CD3/CD28‐stimulated CD8^+^ T cells (L, *n* = 3) toward PIF from TMPD‐treated WT and *Ebi2*‐cKO mice or toward CXCL10 (100 ng mL^−1^). Data are shown as mean ± SEM or representative from three independent experiments (E,F,I,K). *p < 0.05 and **p < 0.01, using a two‐tailed, unpaired Student's *t*‐test (C–E [right panel], G,H,J), or one‐way ANOVA with Holm–Sidak's multiple comparisons test (L).

To investigate the in vivo role of EBI2 in lupus, we constructed TMPD‐induced lupus model in WT and *Ebi2*‐cKO mice. After 2 weeks of induction, peritoneal monocytes were sorted for RNA‐seq (Figure S9A,B, Supporting Information), and the global gene expression profile identified 267 DEGs (*P* < 0.05) including 110 up‐regulated and 157 down‐regulated genes in *Ebi2*‐cKO monocytes. The substantially reduced *Gpr183* expression confirmed the KO efficiency (**Figure** **6**A). *Ebi2*‐cKO monocytes strikingly increased expression of chemokines and cytokines such as *Cxcl10*, *Cxcl11*, *Ccl2*, *Ccl12*, and *Il6*, whereas *Tgfb3* expression was down‐regulated (Figure 6A). Intriguingly, serum concentrations of these up‐regulated genes in *Ebi2*‐cKO monocytes were previously reported to be increased in SLE patients,^[^ ^30^ ^]^ and TGF‐*β*3 was reported to negatively regulate SLE.^[^ ^31^ ^]^ Kyoto Encyclopedia of Genes and Genomes (KEGG) pathway enrichment analysis also revealed that cytokine and cytokine receptor interaction were the top two enriched signaling pathways (Figure 6B). Next, we substantiated the increased production of *Cxcl10*, *Cxcl11*, *Ccl2*, and *Ccl12* in TMPD‐treated *Ebi2*‐cKO monocytes (Figure 6C), and CXCL10 and CCL2 in PIF of *Ebi2*‐cKO mice (Figure 6D). Also, several genes related to SLE, including *Ifit2*, *Ifi208*, and *Tlr9*, were increased in *Ebi2*‐cKO monocytes (Figure S9C, Supporting Information).

EBI2 has been reported to enhance immune cell migration to accelerate the development of multiple sclerosis.^[^ ^16^ ^]^ However, we surprisingly observed that the numbers of immune cells were robustly increased in the peritoneal cavity of *Ebi2*‐cKO mice upon TMPD administration (Figure 6E). Not only the elevated percentages of monocytes (Ly6C^hi^Ly6G^−^), but also the increased numbers of neutrophil (Ly6C^hi^Ly6G^+^), monocytes, CD8^+^ T cells, and B cells were observed in the peritoneal cavity of *Ebi2*‐cKO mice (Figure 6F–H). Moreover, the percentages and numbers of CD4^+^ and CD8^+^ T cells were decreased in spleen of TMPD‐treated *Ebi2*‐cKO mice (Figure 6I,J). To further investigate whether the enhanced immune cell infiltration was associated with the elevated chemokines in PIF of TMPD‐treated *Ebi2*‐cKO mice, we performed a Transwell migration assay and found that PIF from *Ebi2*‐cKO mice attracted more PEMs or CD8^+^ T cell toward the bottom than PIF from WT mice (Figure 6K,L and Figure S9D, Supporting Information).

It is possible that the augmented expression of chemokine receptors or enhanced cell proliferation may also contribute to cell accumulation in the peritoneal cavity of *Ebi2*‐cKO mice. However, we found that transcription levels of *Cxcr3* (receptor for CXCL10 and CXCL11), *Cxcr7* (receptor for CXCL11), and *Ccr2* (receptor for CCL2) were comparable in WT and *Ebi2*‐cKO peritoneal cells at 2 or 12 weeks after TMPD induction (Figure S10A, Supporting Information). Moreover, mRNA levels of *Ccr2* in sorted peritoneal monocytes or *Cxcr3* and *Cxcr7* in sorted peritoneal T cells, and surface CXCR3 expression in splenic or peritoneal T cells remained unchanged in TMPD‐treated *Ebi2*‐cKO mice (Figure S10B–E, Supporting Information). Next, we detected the in vivo proliferation of myeloid cells, T cells, and B cells with EdU staining, which was all unaffected in *Ebi2*‐cKO mice (Figure S10F–J, Supporting Information). Collectively, we suggest that *Ebi2*‐cKO macrophages/myeloid cells enhance chemokine production to shape the microenvironment, which remarkably promotes migration of myeloid and T cells to overcome the effect of EBI2 deficiency on cell migration.

### 2.7 *Ebi2*‐cKO Lupus Mice Show the Enhanced Lymphocyte Activation

**Figure 7:**
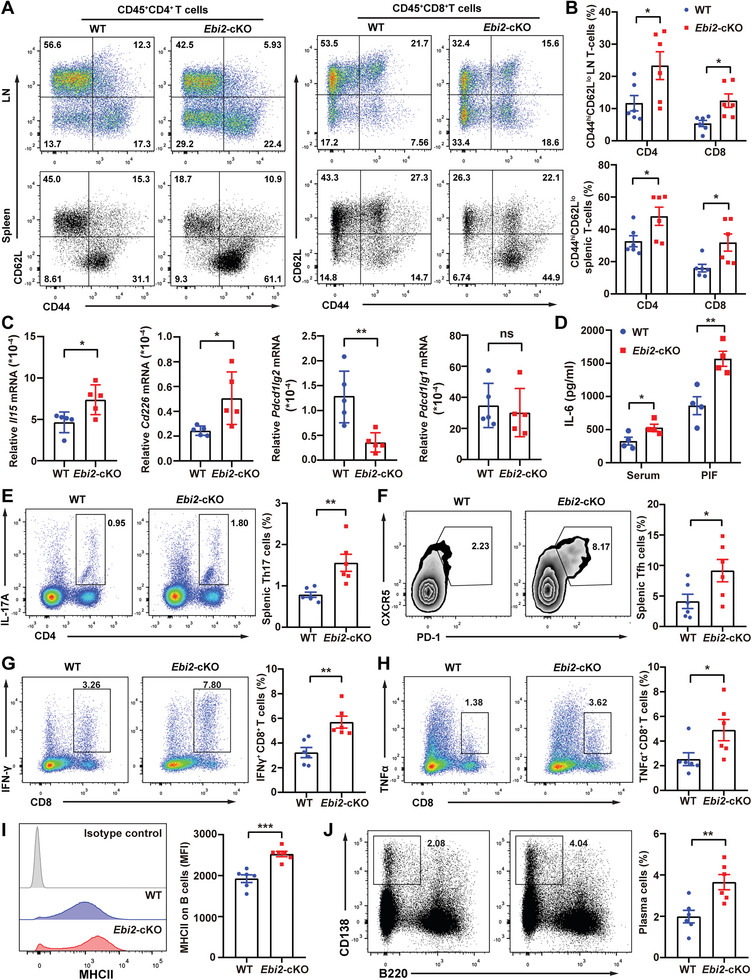
*Ebi2*‐cKO lupus mice show the enhanced lymphocyte activation. A,B,D–J) WT and *Ebi2*‐cKO mice were treated with TMPD for 12 weeks. Representative dot plots of naïve (CD44^lo^CD62L^hi^), central memory (CD44^hi^CD62L^hi^), and effector memory (CD44^hi^CD62L^lo^) T cell subsets in LN and spleen (A), statistical analysis of the percentages of effector memory T cells from LN or spleen (B, *n* = 6), IL‐6 concentrations in serum and PIF (D, *n* = 4), flow cytometric analysis of CD4^+^IL‐17A^+^ T_H_17 cells (E, *n* = 6) or CD4^+^PD‐1^+^ CXCR5^+^ T_FH_ cells (F, *n* = 6) in spleen, the production of IFN‐*γ* and TNF‐*α* in splenic CD8^+^ T cells (G,H, *n* = 6), MHCII expression on CD19^+^ B cells (I, *n* = 6) and the percentage of plasma cells (J, *n* = 6) in spleen. C) WT (*n* = 5) and *Ebi2*‐cKO (*n* = 5) mice were treated with TMPD for 2 weeks to detect the mRNA expression of *Il15*, *Cd226*, *Pdcd1lg2*, and *Pdcd1lg1* in peritoneal monocytes. Data are shown as mean ± SEM or representative from three independent experiments (A,E–J [left panels]). *p < 0.05, **p < 0.01, and ***p < 0.001, using a two‐tailed, unpaired Student's *t*‐test (B–D, and E–J [right panels]).

Abnormal activation of T and B cells is a hallmark of SLE, and macrophages can secret cytokines and present autoantigens to regulate lymphocyte activation.^[^ ^32^ ^]^ We next investigated whether EBI2‐deficient macrophages affect the adaptive immune response. Flow cytometric analysis showed increased percentages of effector memory (CD44^hi^ CD62L^lo^) CD4^+^ and CD8^+^ T cells as well as decreased percentages of naïve (CD44^lo^ CD62L^hi^) T cells in lymph nodes (LNs) and spleens of *Ebi2*‐cKO mice (**Figure** **7**A,B and Figure S11A,B, Supporting Information). To explore how EBI2‐deficient macrophages promote T cell activation, we analyzed the RNA‐seq data and found several up‐regulated DEGs such as *Il15*, *Il6*, and *Cd226* and down‐regulated DEG *pdcd1lg2* in *Ebi2*‐cKO monocytes (Figure S11C, Supporting Information). We confirmed these changes in TMPD‐treated *Ebi2*‐cKO monocytes or ISD‐stimulated *Ebi2*‐cKO PEMs (Figure 7C and Figure S11D, Supporting Information). In addition to the decreased expression of PD‐L2 may weaken the inhibition of T cell activation,^[^ ^33^ ^]^ the increased IL‐15 could also promote the proliferation of CD4^+^ effector memory T cells and support both central and effector memory CD8^+^ T cells.^[^ ^34^ ^]^ Besides, IL‐6 concentrations in serum and PIF (Figure 7D), or *Il1b* transcription levels in peritoneal cells (Figure S11E, Supporting Information) were increased in *Ebi2*‐cKO mice. IL‐6 is involved in T_FH_ differentiation,^[^ ^35^ ^]^ and IL‐6 or IL‐1*β* is an important driver for T_H_17 differentiation.^[^ ^36^ ^]^ Indeed, the frequencies of splenic T_H_17 (CD4^+^IL‐17A^+^) cells (Figure 7E) and T_FH_ (CD4^+^CXCR5^+^PD‐1^+^) cells (Figure 7F) were increased in *Ebi2*‐cKO mice. Furthermore, the production of IFN‐*γ* and TNF‐*α* was markedly increased in splenic CD8^+^ T cells of *Ebi2*‐cKO mice (Figure 7G,H). These results suggest that EBI2‐deficient macrophages promote the activation of T_H_17, T_FH_, and CD8^+^ T cells via modulating the production of various cytokines during the development of TMPD‐induced SLE.

TGF‐*β* inhibits B cell proliferation, plasma cell differentiation, and antibody production, while IFN‐I, IL‐6, and T_FH_ cells promote these processes.^[^ ^31^, ^37^ ^]^ In agreement with the reduced levels of TGF‐*β* and increased levels of IL‐6, IFN‐I, and percentages of T_FH_ cells in EBI2‐deficient cells or mice (Figure 7D,F and Figure S11F, Supporting Information), we found that *Ebi2*‐cKO lupus mice enhanced surface MHCII levels on B cells (Figure 7I) and increased the percentages of plasma cells (Figure 7J). These findings suggest that EBI2‐deficient macrophages promote the production of cytokines and chemokines, which might activate B cells and plasma cells to accelerate SLE development.

### 2.8 EBI2 Deficiency in Macrophages Aggravates the Lupus Nephritis

**Figure 8:**
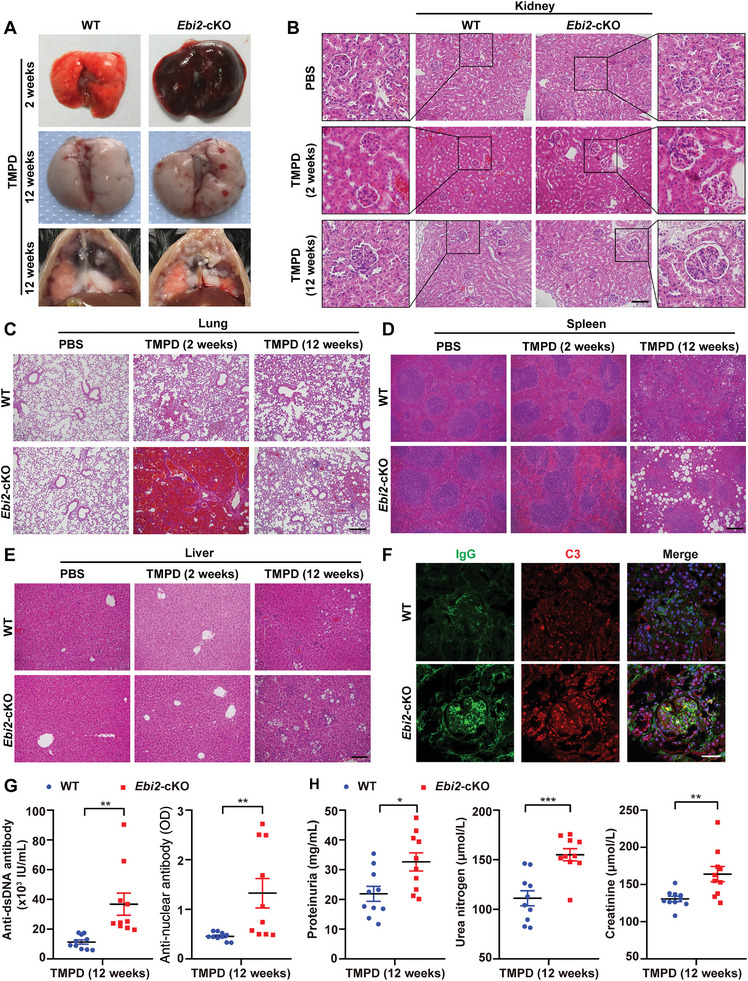
EBI2 deficiency in macrophages aggravates the lupus nephritis. A–H) WT and *Ebi2*‐cKO mice were treated with TMPD for 2 or 12 weeks. Representative images of lungs and lipogranulomas in peritoneal cavity (A) or H&E staining of kidney (B), lung (C), spleen (D), and liver (E, scale bar: 100 µm), or immunofluorescence of glomerular deposition of C3 and IgG (F, scale bar: 30 µm), or serum anti‐dsDNA antibody and anti‐nuclear antibody (G, *n* = 10), or levels of proteinuria, urea nitrogen, and creatinine in urine samples (H, *n* = 10). Scale bars: 200 µm (B–D). Data are shown as mean ± SEM or representative from three independent experiments (A–F). *p < 0.01, **p < 0.01, and ***p < 0.001, using a two‐tailed, unpaired Student's *t*‐test (G,H).

Next, we investigated how EBI2‐deficient macrophages affect the development of SLE in vivo using TMPD‐induced lupus model, which features the development of lipogranulomas adhering to peritoneal mesothelial cell surface and diffuse pulmonary hemorrhage (DPH).^[^ ^23^, ^38^ ^]^ Compared with WT mice, more *Ebi2*‐cKO mice developed complete DPH 2 weeks after TMPD injection and displayed more severe hemorrhagic spots in lung and more lipogranulomas at the end of 12 weeks (**Figure** **8**A). Hematoxylin and eosin (H&E) staining demonstrated worsened histologic damage in *Ebi2*‐cKO mice, as evidenced by enlarged glomerulus and pulmonary hemorrhage (Figure 8B,C), more diffused immune cells in the red pulp of spleen, and more severe vacuolization in spleen and liver (Figure 8D,E). In addition, immunostaining analysis revealed enhanced deposition of immunoglobulin G (IgG) and complement 3 (C3) in the glomeruli of *Ebi2*‐cKO mice (Figure 8F). Consistently, serum levels of anti‐dsDNA antibody and anti‐nuclear antibody (ANA) were significantly increased in *Ebi2*‐cKO mice (Figure 8G). Quantitative analysis of urine samples exhibited elevated levels of proteinuria, urea nitrogen, and creatinine in *Ebi2*‐cKO mice (Figure 8H). These results indicate that EBI2 deficiency in macrophages promotes the development of lupus in mice.

In summary, we propose that the enhanced levels of the ligand 7*α*, 25‐OHC could bind and activate EBI2 to reduce the expression of IFN‐I and ISGs, thus protecting SLE patients. However, during the development of SLE, IFN‐*γ* secreted by activated T cell reduces EBI2 expression in macrophages, resulting in increased production of chemokines and cytokines to not only attract myeloid and T cells, but also enhance lymphocyte activation to worsen SLE pathogenesis. Together, we propose that the GPCR EBI2 acts as a key effector that links the crosstalk between innate and adaptive immunity, and it holds promise as a potential diagnostic marker or therapeutic target for SLE.

## 3 Discussion

EBI2 is a well‐known chemotactic receptor that mediates the migration of various immune cells, including B cells, T cells, dendritic cells, astrocytes, and group 3 innate lymphoid cells, to sites containing the ligand 7*α*, 25‐OHC.^[^ ^16^, ^39^ ^]^ Therefore, we expected that *Ebi2^fl^* ^/^ *^fl^*‐LysM‐Cre mice might exhibit reduced infiltration of myeloid cells during the development of SLE. Surprisingly, after TMPD induction, we found increased infiltration of myeloid cells in the peritoneal cavity of *Ebi2*‐cKO mice, along with enhanced production of IFNs and ISGs such as CXCL10 and CCL2, which suggests that this extrinsic microenvironment could overcome the intrinsic EBI2 deficiency‐mediated reduction of myeloid cell migration. In addition, we observed reduced mRNA and protein expression of EBI2 in PBMCs of SLE patients, as well as in splenocytes, peritoneal cells, and peripheral blood cells of SLE mice. These observations indicate that other immune cells besides monocyte/macrophages may also down‐regulate EBI2 expression during the progression of SLE. Consequently, further research is needed to investigate whether the 7*α*, 25‐OHC/EBI2 signaling in other immune cells with EBI2 down‐regulation is involved in the pathogenesis of SLE.

An integrated genome‐wide association study suggests EBI2 as a negative regulator of the IRF7‐driven inflammatory network, which was associated with susceptibility to type 1 diabetes.^[^ ^40^ ^]^ In line with this, we have elucidated that EBI2 suppresses JAK‐STAT activation via GNAI3, resulting in the reduced ISGs production to limit the progression of SLE. Furthermore, we found that the significantly reduced expression of EBI2 in monocyte/macrophages of SLE patients and mice could be triggered by IFN‐*γ* in a paracrine manner. Since autoantigens‐activated CD4^+^ T cells or CD8^+^ T cells produce IFN‐*γ* during the development of SLE, this leads to the reduction of EBI2 expression in monocyte/macrophages, which alleviates its inhibitory effect on STAT activation to produce higher levels of chemokines and cytokines that not only further facilitates lymphocyte activation and IFN‐*γ* secretion but also promotes differentiation of lymphocytes into T_H_17, T_FH_, and plasma cells, reinforcing the development of SLE. Together, we propose a feedback loop linking monocyte/macrophages and T cells via EBI2 during the progression of SLE. Additionally, we provided a new angle to understand EBI2 function, that is, reducing EBI2 expression in macrophages could enhance IFNs, cytokines and chemokines in the microenvironment, which reshape the innate and adaptive immune responses to promote SLE development.

Although SLE patients exhibit profoundly reduced surface expression of EBI2 on monocytes, patients with RA, DM, or SS display normal EBI2 levels. A previous study has shown that 7*α*, 25‐OHC stimulation could induce *β*‐arrestin‐mediated internalization of EBI2.^[^ ^14b^ ^]^ We observed that the transcription of enzymes for 7*α*, 25‐OHC biosynthesis, including CH25H and CYP7B1, was enhanced in PBMCs and monocytes of SLE patients, which is consistent with the increased plasma concentrations of 7*α*, 25‐OHC in SLE patients. Therefore, the down‐regulated surface expression of EBI2 in SLE patients might be partially due to its internalization and desensitization after sensing the elevated ligand 7*α*, 25‐OHC. Together, EBI2 serves as a key receptor for macrophages to sense cholesterol metabolites in the microenvironment, which could modulate cytokine production to reshape the microenvironment or the function of innate and adaptive immune cells. These characters make EBI2 a precisely controlled GPCR at its expression and signaling levels by IFN‐related regulators (i.e., IFN‐*γ*, oxysterol, STAT, IFN‐I, and ISGs) during the development of SLE.

We have found that both the expression levels of plasma 7*α*, 25‐OHC and EBI2 on the surface of monocyte/macrophage may serve as diagnostic markers to predict the outcome of SLE. Moreover, as GPCRs are favorable drug targets, exploring potential agonists to maintain EBI2 surface expression might provide new therapeutic strategies for SLE. Given that the expression and signaling of EBI2 are controlled by multiple IFN‐related regulators (i.e., IFN‐*γ*, oxysterol, STAT, IFN‐I, and ISGs), it would be exciting to investigate whether detecting the surface expression of EBI2 could predict the therapeutic efficacy of SLE patients who receive Anifrolumab treatment.

## 4 Experimental Section

### Mice

The second exon of *Ebi2* gene was flanked by two loxP sites to generate *Ebi2* genetically ablated mice (Beijing Biocytogen Co., Ltd.). *Ebi2^fl^* ^/−^ mice were crossbred with *Lyz2*‐*Cre* ^+/+^ mice to generate *Ebi2^fl^* ^/−^ *Lyz2*‐*Cre* ^+/−^ mice, which were then backcrossed with *Ebi2^fl^* ^/−^ mice. *Ebi2^fl^* ^/^ *^fl^Lyz2*‐*Cre* ^+/−^ or *Ebi2^fl^* ^/^ *^fl^Lyz2*‐*Cre* ^+/+^ (termed *Ebi2*‐cKO) mice specifically knockout *Ebi2* in myeloid cells, and the littermate *Ebi2^fl^* ^/^ *^fl^Lyz2*‐*Cre* ^−/−^ (termed WT) were used as the controls. Genotyping was performed using the primers: forward, 5′‐ACTTTCTGGGTTGACACAGATCAGATT‐3′; reverse, 5′‐TACAGTTGGTGGCTTTGGCAGAACT‐3′. *Gnai3* ^−/−^ mice were kindly provided by Dr. Beicheng Sun (The Affiliated Drum Tower Hospital of Nanjing University Medical School). All mice were C57BL/6 background, and were randomly allocated to experimental groups with the same age (8–12 weeks) and sex. Mice were bred under specific pathogen‐free conditions and animal studies were approved by the Animal Care Facility of Shanghai Institute of Biochemistry and Cell Biology (Protocol No. IBCB0057), Chinese Academy of Science.

### Antibodies and Reagents

Antibodies recognizing p‐STAT1 (Tyr701 clone 58D6) (#9167), STAT1 (D1K9Y) (#14994), p‐STAT2 (Tyr690 clone D3P2P) (#88410), STAT2 (D9J7L) (#72604), p‐STAT3 (Tyr705 clone D3A7) (#9145), STAT3 (124H6) (#9139), p‐IRF3 (Ser 396 clone 4D4G) (#4974), IRF3 (D83B9) (#4302), p‐TBK1 (Ser172 clone D52C2) (#5483), TBK1 (clone D1B4) (#3504), p‐p38 MAPK (Thr180/Tyr182 clone D3F9) (#4511), p38 MAPK (D13E1) (#8690), p‐p44/42 MAPK (Erk1/2) (Thr202/Tyr204 clone D13.14.4E) (#4370), p44/42 MAPK (Erk1/2) (137F5) (#4695), and Myc (71D10) (#2278) were obtained from Cell Signaling Technology. Anti‐EBI2 polyclonal antibody (sc‐6640) was purchased from Santa Cruz. Horseradish peroxidase (HRP)‐conjugated GAPDH monoclonal antibody (HRP‐60004) and anti‐C3/C3b/C3c polyclonal antibody (21337‐1‐AP) were purchased from Proteintech. Anti‐EBI2 antibody (ab168744) and HRP‐*β*‐actin (ab49900) antibodies were obtained from Abcam. Monoclonal anti‐HA (H3663), poly(deoxyadenylic‐thymidylic) acid sodium salt (86828‐69‐5), 7*α*, 25‐OHC (64907‐22‐8), and pristane (1921‐70‐6) were purchased from Sigma. Secondary HRP‐labeled antibodies against rabbit (A0208) or mouse (A0216) and BCA protein assay kit (P0012) were obtained from Beyotime. PE anti‐human GPR183 (368912) and PE anti‐mouse IgG2a, *κ* Isotype Ctrl (400212), APC‐cyanine 7 anti‐mouse F4/80 (123118), and Brilliant Violet 421 anti‐CD45 (103133) antibodies were purchased from BioLegend. PerCP‐cyanine 5.5 anti‐human CD19 (45‐0199), FITC anti‐human CD14 (11‐0149), APC anti‐human CD11b (17‐0118), FITC anti‐human CD4 (11‐0049), APC anti‐human CD8a (17‐0087), FITC anti‐mouse CD11b (11‐0112), FITC anti‐mouse B220 (11‐0452), FITC anti‐mouse CD3e (11‐0031), APC anti‐mouse CD8a (17‐0081), PE anti‐mouse CD4 (12‐0041), FITC anti‐mouse Ly‐6G (11‐9668), APC anti‐mouse Ly‐6C (17‐5932), PE‐cyanine 7 anti‐mouse CD11c (25‐0114), PE anti‐mouse CD138 (281‐2), PerCP‐cyanine 5.5 anti‐mouse CD19 (45‐0193), APC anti‐mouse IFN‐*γ* (17‐7311), PE anti‐mouse IL‐17a (12‐7177), PE anti‐mouse TNF‐*α* (12‐7321), APC anti‐mouse CD183 (CXCR3) (17‐1831), Fixable Viability Dye eFluor 506 (65‐0866‐18) antibodies, and Rat anti‐mouse IFN gamma monoclonal antibody (R4‐6A2) (MM701) were obtained from eBioscience. PE anti‐mouse I‐A[b] (553552) antibody was obtained from BD Pharmingen. Lymphocyte separation medium (LSM, 0850494) was purchased from MP Biomedicals. CpG‐B (ODN1668) (tlrl‐1668‐1), R848 (resiquimod) (tlrl‐r848), imiquimod (R837) (tlrl‐imqs), and poly (I:C) (HMW) (tlrl‐pic) were purchased from InvivoGen. Polybrene transfection regent (TR‐1003‐G) was purchased from Merck Millipore. ANA Hep Screen ELISA (DE7020) was purchased from Demeditec. Anti‐dsDNA enzyme immunoassay test kit (0104000143) was obtained from Shanghai Kexin Biotech Co., Ltd. Urea nitrogen (BUN) detection Kit (JN‐025698) was purchased from Shanghai Jining Industrial Co., Ltd. Creatinine assay kit (Colorimetric) (ab204537) was purchased from Abcam. Mouse IP‐10/CXCL10 ELISA Kit (F10933) and mouse MCP‐1/CCL2 ELISA Kit (F11130) were obtained from Shanghai Xitang Biotech Co., Ltd. Mouse IFN‐*β* ELISA Kit (42400) was purchased from PBL Assay Science. Mouse IL‐6 ELISA Kit (85‐88‐7964‐89) was purchased from eBioscience. Q‐VD‐OPh (HY‐12305) and ABT‐737 (HY‐50907) were purchased from MCE.

### Immunoprecipitation and Immunoblotting Assay

HEK293T cells or iBMDMs were washed with ice‐cold PBS and lysed with RIPA lysis buffer (50 mm Tris‐HCl, 150 mM NaCl, 1% Nonidet P40, 0.1% SDS, 1 mm EDTA, and 0.25% sodium‐deoxycholate) supplemented with protease inhibitors NaF, Na_3_VO_4_, PMSF, and cocktails. Cell lysates were incubated with indicated antibodies, followed by protein G Sepharose beads incubation at 4 °C. The Sepharose beads were then washed three to five times with lysis buffer and resuspended in an appropriate amount of SDS‐PAGE loading buffer. The samples were separated by 8–10% SDS‐PAGE gel, followed by immunoblotting analysis with the indicated antibodies.

### Plasmids and Transfection

Human *EBI2* was amplified from pcDNA3.1‐EBI2 plasmid (a gift from Dr. Hongwen Chen) and was cloned into pUltra vector or MIGR‐IRES‐GFP vector with N‐terminal FLAG tag, respectively. Mouse *Gnai3* was amplified from reverse‐transcribed cDNA of PEMs and was cloned into the pcDNA3.1 vector with a C‐terminal HA tag. Human JAK1, JAK2, JAK3, and TYK2 cDNA were obtained from the 293T cell line and inserted into the MIGR‐IRES‐GFP vector with C‐terminal HA tag. pUltra‐EBI2 plasmids were transfected with the lentiviral packaging plasmids PASPX and pMD2 to HEK293T cells. Lentiviral supernatants were collected to infect iBMDMs, followed by GFP^+^ cell sorting to generate stably transfected cells. For replenishing hEBI2 in primary *Ebi2*‐cKO BMDMs, BMs from WT and *Ebi2*‐cKO mice were collected and cultured with complete DMEM supplemented with recombinant mouse M‐CSF (25 ng µL^−1^) (Novoprotein, CK02) for 2 days, then infected with retrovirus supernatants supplemented with M‐CSF (25 ng mL^−1^) and polybrene (10 µg mL^−1^) from MIGR‐EBI2 or MEGR‐GFP transfected platinum‐E (Plat‐E) cells every 12 h for 3 days. After infection, cells were again cultured with complete DMEM for another 12 h before ISD stimulation.

### Cell Culture

PEMs were harvested from mice with intraperitoneal (i.p.) injection of 3% brewer thioglycollate medium (3 mL) for 4 days and then cultured in complete DMEM. BMDMs were generated after culturing BM cells for a week with complete DMEM containing M‐CSF (20 ng µL^−1^). PEMs, BMDMs, iBMDMs, HEK293T, and plat‐E cells were cultured in complete DMEM supplemented with 10% v/v FBS, 1% v/v glutamine, and 1% penicillin/streptomycin (100 U mL^−1^) at 37 °C with 5% CO_2_.

### LC‐MS Analysis of Sterols in Plasma and Peritoneal Irrigation Fluid

Quantitative analysis of sterols including cholesterol, oxysterols, and steroid hormones was done using a reported LC‐MS/MS method^[^ ^41^ ^]^ with some modifications.^[^ ^42^ ^]^ In brief, plasma (50 µL) was mixed with pre‐cooled methanol‐water (1 mL, 10:1, −20 °C) and methanol solution (10 µL) of internal standards including cholesterol‐d7, 24‐hydroxycholesterol‐d7, campesterol‐d3, estrone‐d2, estradiol‐d2, dehydroepiandrosterone‐d2, and *β*‐sitosterol‐d7 (Steraloids, USA). After 10 min centrifugation (12 000 rpm, 4 °C), supernatant was obtained and dried with nitrogen gas to obtain extract residues. The extract residues from each sample were, respectively, added with pyridine solution (100 µL) of derivatization reagents consisting of 2‐picolinic acid (8 mg), 2‐methyl‐6‐nitrobenzonic anhydride (22 mg), and dimethylaminopyridine (4 mg) from Aladdin (Shanghai, China). After standing at 80 °C for 60 min, the mixture was added with water (200 µL) and extracted with MTBE (1.5 mL). The upper layer was dried under nitrogen gas and then re‐dissolved in acetonitrile (100 µL) for LC‐MS/MS analysis.

LC‐MS/MS analysis was conducted on an UHPLC‐MS system with a 30AD UHPLC (Shimadzu, Japan) and a QTRAP 6500plus mass spectrometer (SCIEX, USA). All data were acquired using Analyst (V1.7, Sciex) and processed with OS (V2.0, Sciex). The chromatographic separation was performed on a Zorbax Eclipse Plus C18 column (100 × 2.1 mm, 1.8 µm, Agilent, USA) with injection volume of 5 µL; gradient elution was done with water (A) and acetonitrile (B) containing 0.1% formic acid in both. The optimized mass spectrometer parameters for electrospray ionization source were employed including ion‐spray voltage (5500 V), curtain gas (40 psi), and source temperature (550 °C). Quantification of all detectable sterols was achieved using these internal standards based on method validation.^[^ ^42^ ^]^

### The Selection of Human Subjects

The current study was approved by the Ethics Committee of Renji Hospital, Shanghai Jiao Tong University (Approval No. KY2022‐023‐B) and Shanghai Tenth People's Hospital, Tongji University (Approval No. SHYS‐IEC‐5.0/22K234/P01). All SLE patients fulfilled the 1997 American College of Rheumatology (ACR) classification criteria for SLE. Patients with DM, RA, and SS were also included as disease controls. DM patients fulfilled the Bohan and Peter's criteria. RA patients fulfilled the 2010 ACR/EULAR RA classification criteria. SS patients fulfilled the 2017 ACR/EULAR classification criteria for primary SS. All HCs had no history of autoimmune diseases. All study subjects were enrolled in the study after giving informed and written consent. Information of SLE patients, disease controls, and healthy donors were listed in Tables S1 and S2, Supporting Information.

### Purification and Fractionation of Human PBMCs

The peripheral blood of healthy donors and SLE patients were collected in anticoagulation tubes, diluted with PBS, and gently layered over an equal volume of LSM in a conical tube and centrifuged at 2200 rpm for 25 min in a swinging bucket rotor without brake. After density gradient centrifugation, four layers formed, and the second layer containing mononuclear cells was carefully transferred into a new conical tube, filled with PBS and centrifuged at 1200 rpm for 10 min. After aspirating the supernatant, isolated PBMCs were immunostained for flow cytometric assay or were resuspended in Trizol reagent (Takara)for RNA extraction. The remaining PBMCs were further fractionated into T‐lymphocytes and monocytes by a T cell negative selection kit (#17951, STEMCELL Technologies) and a CD14 positive selection kit (#17818, STEMCELL Technologies). Briefly, PBMCs were mixed with the selection cocktails (5 µL per 10^7^ cells) and incubation at room temperature (RT) for 5 min. After adding recommend medium to top up the sample to the indicated volume, the mixtures were subsequently placed into the magnet to acquire the supernatant or attached beads. The purified cells were resuspended in Trizol reagent for RNA extraction. For human CD3^+^ T cells induction, 48‐well plate was pre‐coated with anti‐CD3 (5 µg mL^−1^) and anti‐CD28 (5 µg mL^−1^) antibodies at 4 °C overnight, then discarded the coating buffer and washed with PBS for three times. CD3^+^ T cells were seeded at 1 × 10^6^ cells/well in 48‐well plate for 24 h, and the supernatant (CD3 conditional medium, CD3 C.M) was collected to stimulated human CD14^+^ monocytes.

### RNA Interference, RNA Extraction, and Real‐Time Fluorescence Quantitative PCR

RNA interference was performed with single siRNA or a mixture of two different siRNAs for one gene. siRNA oligonucleotides of *Jak1*, *Jak2*, *Stat1*, *Stat2*, *Ebi2*, *Gani1*, *Gani2*, *Gani3*, and *Gnas* were all designed by siRNA selection program and synthesized from GenePharma. Murine PEMs were transfected with siRNA (40 nm) using Lipofectamine RNAiMAX reagent (Invitrogen). The siRNA sequences are shown in Table S3, Supporting Information. Total RNA was extracted with Trizol reagent, and cDNA was obtained with reverse transcriptase M‐MLV (Takara) for quantitative real‐time PCR (qRT‐PCR) using SYBR Green Dye (2043, DBI Bioscience) on a LightCycler480 (Roche). The primers for qRT‐PCR are shown in Table S4, Supporting Information.

### TMPD‐Induced IFN Signature and Nephritis

To induce IFN signature, 8‐week‐old mice (WT and *Ebi2*‐cKO) received a single i.p. injection of TMPD (0.5 mL) as previously described. Control mice received PBS (0.5 mL) at the same time. Female mice were selected for the study because they were more susceptible to the disease. 2 weeks after injection, mice were euthanized and peritoneal cavities were washed with PBS (5 mL) containing EDTA (2 mm) to harvest total peritoneal cells. For TMPD‐induced nephritis, mice were given TMPD by i.p. injection (0.5 mL per mouse) for 12 weeks. The urine of experimental animals was then collected for proteinuria, creatinine, and urea nitrogen analysis using BCA protein assay kit, creatinine assay kit, and urea nitrogen detection kit according to the manufacturer's instructions, respectively. The peripheral blood cells, peritoneal cells, LN cells, and splenocytes were collected for flow cytometric assay. The kidney, spleen, lung, and liver tissues of mice were collected after TMPD injection for 2 and 12 weeks to perform H&E staining or immunofluorescence assay. All animal procedures were conducted in strict accordance with the institutional guidelines and were approved by the Institutional Animal Care and Use Committee of Shanghai Institute of Biochemistry and Cell Biology (IBCB0057).

### ELISA

Concentrations of IL‐6, CXCL10, and CCL2 in serum or PIF of TMPD‐treated mice, or IFN‐*β* in cell culture supernatants were measured by commercial ELISA kits according to the manufacturer's instructions.

### Histopathological and Immunofluorescence Analysis

For histopathological analysis, the kidney, spleen, lung, and liver tissues of TMPD‐treated mice were fixed in 4% paraformaldehyde (PFA) and embedded in paraffin. After serially cutting, the tissue sections were stained with H&E. The pathological changes were evaluated by BX51 light microscope (Olympus). For immunofluorescence analysis, kidney tissues were fixed in 4% PFA and dehydrated with 30% sucrose, and embedded in OCT compound. Kidney slices (10 µm in thickness) were cut using microtome (Leica), and blocked with PBS containing 5% donkey serum for 30 min at RT, and then incubated with anti‐C3 antibody at appropriate final dilutions in a humidified chamber at RT for 2 h. After washing with PBS for three times, the tissue slices were incubated with Cy3 donkey anti‐rabbit and F488 donkey anti‐mouse antibodies for 1 h, followed by staining the nucleus with DAPI for 10 min at RT. Tissue slices were mounted and then examined using a Leica TCS SP8 confocal microscope. Spleen slices (4 µm in thickness) from paraffin‐embedded tissues was stained with anti‐EBI2 antibody at RT for 2 h, followed by F488 donkey anti‐mouse antibody and DAPI staining for 1 h to detect EBI2 expression.

### CD8^+^ T Cells Migration Assay

Naïve CD8^+^ T cells were isolated from spleens of WT mice with BD IMag anti‐mouse CD8a particles and stimulated with anti‐CD3 (2 µg mL^−1^) and anti‐CD28 (2 µg mL^−1^) for 12 h. CD8^+^ T cells were collected and washed by migration media (HBSS with 0.1% protease‐free BSA), and adjusted volume to 5 × 10^5^ cells/100 µL. Transwell inserts were removed from the 24 well plates (Corning costar, 3421), and migration media (600 µL) was added with recombinant human CXCL10 (100 ng mL^−1^) (Novoprotein, P02778) or conditional medium (the ratio of complete RPMI1640 medium and PIF of TMPD‐treated WT or *Ebi2*‐cKO mice was 2:1). The Transwell inserts were placed onto the receiving plates and CD8^+^ T cells (100 µL) were added into the inserts to observe the migration of T cells every 1 h. The inserts were removed at proper time, and Hoechst 33342 was added to the receiving plates. The cell number in the receiving plates was recorded by Operetta (PerkinElmer).

### PEMs Migration Assay

For PEMs migration, PEMs (2 × 10^5^) were seeded in the top of the Transwell inserts (Corning costar, 3422) in a complete DMEM, and conditional medium (the ratio of complete DMEM and PIF of TMPD‐treated WT or *Ebi2*‐cKO mice was 2:1) was added in the well below. After incubation for 6 h, the inserts were washed using 1× PBS and the cells were fixed with 100% methanol for 15 min, followed by cell staining using 0.5% crystal violet (filtered) for 30 min. The upper membrane of the inserts was next cleaned to remove excess stain and non‐migrated cells. Five microscopic fields of each insert (one field in the center and four fields in the periphery of the membrane) were captured by BX51 light microscope (Olympus).

### RNA Sequencing Analysis

Monocytes (CD11b^+^Ly6c^+^ cells) were sorted from peritoneal cells of WT or *Ebi2*‐cKO mice with TMPD treatment for 2 weeks. Total RNA was extracted with Trizol reagent (Invitrogen) and subjected to RNA‐seq analysis using an Illumina HiSeq‐X‐ten sequencer in Shanghai Majorbio Bio‐Pharm Technology Co., Ltd. Hisat2 was used for genome mapping, and the mapping ratio was over 90% across all the samples in the dataset. Fragments per kilobase of exon model per million mapped reads (FPKM) were used to normalize gene expression. StringTie was run with FPKM values for known gene models. edgeR was used to identify DEGs. The *p*‐value significance threshold in multiple tests was set by the false discovery rate (FDR). The fold‐changes were estimated according to the FPKM in each sample. The DEGs were selected using the following filter criteria: FDR ≤ 0.05 and fold‐change ≥ 2.

### Statistical Analysis

All sample sizes were large enough to ensure proper statistical analysis. Data were represented as the mean ± SEM from at least three different experiments. Statistical analysis was performed using GraphPad Prism8 software, version 8 (GraphPad Software, Inc.). Statistical significance was calculated using Student's two‐tailed unpaired *t*‐test or ANOVA with Holm–Sidak multiple comparisons test. Correlations were done using Pearson's test. ns, no significance (*p* > 0.05). *p* < 0.05 was considered statistically significant (*p < 0.05, **p < 0.01, ***p < 0.001, and ****p < 0.0001).

## Conflict of Interest

The authors declare no conflict of interest.

## Author Contributions

F.Z., B.Z., and H.D. contributed equally to this work. B.W., H.W., and F.Z. designed research. F.Z. and B.Z. performed the majority experiments and statistical analysis. H.D. and N.S. provided the blood samples and information of patients with SLE, RA, SS, and DM and offered professional advice. Xiang.L., X.W., Xia.Z., Q.L., Q.F., L.Q., D.Y., Xiao.L., Xin.Z., and Q.Z. participated in part of the experiments. M.H. contributed the scRNA‐seq analysis data. L.C. and H.T. detected cholesterol and its derivatives in plasma of SLE patients by LC‐MS. J.Q. and Z.Z. detected cholesterol and its derivatives in peritoneal lavage fluid of lupus mice by LC‐MS. F.Z. drafted the manuscript. B.W.,H.W.,F.Z., B.Z., and Xiang.L.reviewed and edited the manuscript. All authors read and approved the final manuscript.

## Supporting information

Supporting Information

## Data Availability

The data that support the findings of this study are available from the corresponding author upon reasonable request.
