# Supplementary material for: The Oxysterol Receptor EBI2 Links Innate and Adaptive Immunity to Limit IFN Response and Systemic Lupus Erythematosus
Source: Adv Sci (Weinh). 2023 Jul 19;10(27):2207108. doi: 10.1002/advs.202207108 (PMC10520634; doi:10.1002/advs.202207108)
Supplement: Supplementary file 1 — Supporting Information [file ADVS-10-2207108-s001.pdf]

ADVANCED SCIENCE
Open Access

Supporting Information

for *Adv. Sci.*, DOI 10.1002/advs.202207108

The Oxysterol Receptor EBI2 Links Innate and Adaptive Immunity to Limit IFN Response and Systemic Lupus Erythematosus

Fang Zhang, Baokai Zhang, Huihua Ding, Xiangyue Li, Xilin Wang, Xiaomin Zhang, Qiaojie Liu, Qiuyun Feng, Mingshun Han, Longlong Chen, Linlin Qi, Dan Yang, Xiaojing Li, Xingguo Zhu, Qi Zhao, Jiaqian Qiu, Zhengjiang Zhu, Huiru Tang, Nan Shen, Hongyan Wang* and Bin Wei**

Supporting Information

The Oxysterol Receptor EBI2 Links Innate and Adaptive Immunity to Limit IFN Response and Systemic Lupus Erythematosus

Fang Zhang[1,2,3,#], Baokai Zhang[1,2,#], Huihua Ding[4,#], Xiangyue Li[1,2], Xilin Wang[1,2], Xiaomin Zhang[5], Qiaojie Liu[5], Qiuyun Feng[1,2], Mingshun Han[6], Longlong Chen[7], Linlin Qi[1,2], Dan Yang[5], Xiaojing Li[1,2], Xingguo Zhu[1,2], Qi Zhao[1,2], Jiaqian Qiu[8], Zhengjiang Zhu[8], Huiru Tang[7], Nan Shen[4*], Hongyan Wang[6,9*], Bin Wei[1,2,3,5,10*]

Supplementary materials and methods

Table S1. Characteristics of SLE, DM, RA, SS patients and healthy controls (HCs).

	SLE	DM	RA	SS	HCs
Sex (Female : male)	35 : 1	19 : 2	5 : 3	7 : 1	37 : 4
Age (year)	38 ± 16	49 ± 14.7	53.5 ± 10.4	53.5 ± 19.2	39 ± 12
SLEDAI	11 ± 6.8				
Disease course (month)	94 ± 94				
24h Urine protein (g)	1670 ± 2111				
C3 concentration (g/L)	0.69 ± 0.38				
C4 concentration (g/L)	0.13 ± 0.10				
Anti-dsDNA (IU/ml)	61.25 ± 49.79				
Anti-nuclear Antibody positive	29 (81 %)				
IgG	15.98 ± 8.92				
IgA	2.52 ± 1.51				
IgM	0.84 ± 0.65				
**Clinical presentation at blood collection**					
Fever	13 (36.1%)				
Rash	16 (44.4%)				
Alopecia	8 (22.2%)				
Arthritis	11 (30.6%)				
Oral ulcer	4 (11.1%)				
Photosensitivity	4 (11.1%)				
Hematological disorder	15 (41.7%)				
Neurological disorder	6 (16.7%)				
**Immunomodulating drugs at blood collection**					
Mycophenolate (MMF)	6 (16.7%)				
Methotrexate	1 (2.8%)				
Cyclophosphamide (CTX)	1 (2.8%)				
Antimalarials	14 (38.9%)				
Prednisone	29 (80.5%)				

Methylprednisolone (MP)	6 (16.7%)				
FK506	2 (5.6%)				
Belimumab	1 (2.8%)				

The continuous variables were presented as the mean ± standard deviation.

Table S2. Detailed information on disease status at the time of blood collection, medication and treatment duration one month prior to blood collection for each SLE patients.

	Disease status at blood collection	Glucocorticoids	Antimalarials	Immunosuppressant
P1	Active - Relapse	Pred 20mg qd*1M	HCQ 0.2g bid*1M	
P2	Active - Inception	MP 80mg*3d → 60mg*2d → 40mg*q12h*2d		CTX 0.8g iv once
P3	Active - Relapse	Pred 15mg qd*3M	HCQ 0.2g qd*1M	MMF 0.75g bid * 3M
P4	Active - Relapse	Pred 12.5mg/d * 1M → 30mg qd*7day		
P5	Active - Relapse	Pred 30mg*7d		
P6	Active - Relapse	MP 40mg qd *3d→Dex 10mg q12h*4d		
P7	Active - Relapse	Pred 15mg bid*1M		
P8	Active - Relapse	Pred 5mg tid*1M	HCQ 0.2g qd*1M	
P9	Active - Relapse	Pred 30mg/d*2M		
P10	Active - Relapse	MP 160mg qd*3d→80mg qd*5d	HCQ 0.1g tid *8d	
P11	Active - Relapse	Pred 40mg qd*17d→30mg qd*13d		MMF 0.75g bid * 1M
P12	Active - Relapse	Pred 15mg qd*16d→40mg qd*14d		
P13	Active - Relapse	Pred 10mg*qd*27d→30mg qd *3d		
P14	Active - Relapse	Pred 20mg qd *13d→ 25mg bid*17d		MMF 1.0g bid *13d → 0.5g bid *17d
P15	Active - Relapse	Pred 40mg*qd*10d	HCQ 0.1g * 10d	
P16	Active - Relapse	Pred 10mg*15d → 50mg*13d		
P17	Active - Relapse	Pred 10mg qd*1M		
P18	Active - Relapse	Pred 5mg qd*1M		
P19	Active - Inception			
P20	Active - Relapse	Pred 25mg qd*1M		MMF 0.75g bid * 1M
P21	Active - Inception	Pred 20mg*qd*22d→20mg bid*6d	HCQ 0.2g qd*1M	
P22	Inactive	Pred 10mg qd*1M	HCQ 0.2g qd*1M	MTX 10mg qw*1M

P23	Inactive	Pred 5mg qd*1M	HCQ 0.2g qd*1M	
P24	Active - Relapse	Pred 25mg bid*1M	HCQ 0.1g bid*1M	FK506 0.5mg bid*1M
P25	Active - Relapse	Pred 15mg qd*1M		MMF 0.75 bid*1M
P26	Active - Inception	MP 40mg*4d	HCQ 0.2g bid*1M	
P27	Active - Relapse	Pred 40mg*1M → MP 40mg*2d		
P28	Active - Relapse	Pred 30mg qd*1M		FK506 0.5mg qd*1M
P29	Active - Inception			
P30	Active - Relapse	MP 40mg qd*16day	HCQ 0.2g bid*1M	
P31	Active - Relapse	Pred 50mg qd*12d → MP 80mg*1d	HCQ 0.2g qd*1M	
P32	Active - Relapse	MP 40mg/d*1 day		
P33	Active - Relapse	Pred 30mg bid*25d	HCQ 0.2g qd*1M	
P34	Active - Relapse	Pred 2.5mg qd*1M		
P35	Active - Relapse		HCQ 0.2g qd*1M	Belimumab 480mg iv once + MMF 0.5 bid*1M
P36	Active - Relapse	Pred 15mg qd*1M		

Table S3. siRNA sequences for knockdown experiments.

Genes	Sense (5'-3')	Anti-sense (5'-3')
Negative Control	UUCUCCGAACGUGUCACGU	ACGUGACACGUUCGGAGAA
Ebi2-1	CUCAACCACUCUCUAUUCA	UGAAUAGAGAGUGGUUGAG
Ebi2-2	CUGCUCUGGUGUUCUACAU	AUGUAGAACACCAGAGCAG
Jak1-1	ACAUUAUGGAGCUGAAAUA	UAUUUCAGCUCCAUAAUGU
Jak1-2	CAUUAUGGAGCUGAAAUAU	AUAUUUCAGCUCCAUAAUG
Jak2-1	UGCUCAAAUGAAAGUAGAA	UUCUACUUUCAUUUGAGCA
Jak2-2	CUCUGUCAGCUACAAGACA	UGUCUUGUAGCUGACAGAG
Stat1-1	ACACUGUGAUGUUAGAUAA	UUAUCUAACAUCACAGUGU
Stat 1-2	CCAUCGAGCUCACUCAGAA	UUCUGAGUGAGCUCGAUGG
Stat2-1	CGACAGGAAUUCAGACUUA	UAAGUCUGAAUUCCUGUCG
Stat 2-2	GAAGGCAGCGAAUCACUCA	UGAGUGAUUCGCUGCCUUC
Gnai1	GGAAGGACACAAAGGAAAU	AUUUCCUUUGUGUCCUUCC
Gnai2	GGACUUACACUUCAAGAUG	CAUCUUGAAGUGUAAGUCC
Gnai3	UCAUUCAUGAGGACGGCUA	UAGCCGUCCUCAUGAAUGA
GNAS-1	UCGAAGAUUGAGGACUACU	AGUAGUCCUCAAUCUUCGA
GNAS-2	CUUUGACUUCCCACCUGAA	UUCAGGUGGGAAGUCAAAG

Table S4. Primers for qRT-PCR.

Genes	Species	Forward Primers (5'-3')	Reverse Primers (5'-3')
Actb	Mouse	GGCTGTATTCCCCTCCATCG	CCAGTTGGTAACAATGCCATGT
Ebi2	Mouse	ATGGCTAACAATTTCACTACCCC	ACCAGCCCAATGATGAAGACC
Dhcr7	Mouse	GCAGGGGTTGTGAACAAGTAT	GAGACGGCATAGCCAAGGAT
Srebp2	Mouse	GCAGCAACGGGACCATTCT	CCCCATGACTAAGTCCTTCAACT
Sqle	Mouse	ATAAGAAATGCGGGGATGTCAC	ATATCCGAGAAGGCAGCGAAC
Hmgcr	Mouse	AGCTTGCCCGAATTGTATGTG	TCTGTTGTGAACCATGTGACTTC
Hmgcs	Mouse	AACTGGTGCAGAAATCTCTAGC	GGTTGAATAGCTCAGAACTAGCC
Idlr	Mouse	TGACTCAGACGAACAAGGCTG	ATCTAGGCAATCTCGGTCTCC

Idol	Mouse	TGCAGGCGTCTAGGGATCAT	ATCTAGGCAATCTCGGTCTCC
Abca1	Mouse	AAAACCGCAGACATCCTTCAG	CATACCGAAACTCGTTCACCC
Abcg1	Mouse	CTTTCCTACTCTGTACCCGAGG	CGGGGCATTCCATTGATAAGG
Acat1	Mouse	GAAACCGGCTGTCAAAATCTGG	TGTGACCATTTCTGTATGTGTCC
Acat2	Mouse	CCCGTGGTCATCGTCTCAG	GGACAGGGCACCATTGAAGG
Ifnb1	Mouse	TACAACAGCTACGCCTGGAT	AGTCCGCCTCTGATGCTTAA
Cxcl10	Mouse	CCAAGTGCTGCCGTCATTTTC	GGCTCGCAGGGATGATTTCAA
Cxcl11	Mouse	GGCTTCCTTATGTTCAAACAGGG	GCCGTTACTCGGGTAAATTACA
Mx1	Mouse	GAAGGCAAGGTCTTGGATG	GCTGACCTCTGCACTTGACT
Mx2	Mouse	GAGGCTCTTCAGAATGAGCAAA	CTCTGCGGTCAGTCTCTCT
Ccl2	Mouse	GCCTGCTGTTCACAGTTGC	CAGGTGAGTGGGGCGTTA
Ifit2	Mouse	AGTACAACGAGTAAGGAGTCACT	AGGCCAGTATGTTGCACATGG
Ifi208	Mouse	TCCCTGAATCACCTGATACCAT	GAGTCAGTGGTTCACCCTCA
Ccl12	Mouse	ATTTCCACACTTCTATGCCTCCT	ATCCAGTATGGTCCTGAAGATCA
Il6	Mouse	TGTATGAACAACGATGATGCACTT	ACTCTGGCTTTGTCTTTCTTGTTATCT
Il1b	Mouse	CTGGTACATCAGCACCTCAC	AGAAACAGTCCAGCCCATAC
Irf7	Mouse	GAGACTGGCTATTGGGGGAG	GACCGAAATGCTTCCAGGG
Il15	Mouse	ACATCCATCTCGTGCTACTTGT	GCCTCTGTTTTAGGGAGACCT
Cd226	Mouse	GACACAACAGTTCGGCTTTCT	TGCCAATGTCTGCTTCTGAGG
Pdxd1lg1	Mouse	GCTCCAAAGGACTTGTACGTG	TGATCTGAAGGGCAGCATTTC
Pdxd1lg2	Mouse	CTGCCGATACTGAACCTGAGC	GCGGTCAAAATCGCACTCC
Tgfb3	Mouse	CCTGGCCCTGCTGAACTTG	TTGATGTGGCCGAAGTCCAAC
Tlr9	Mouse	ATGGTTCTCCGTCGAAGGACT	GAGGCTTCAGCTCACAGGG
Cxcr3	Mouse	AACAGCACCTCTCCCTACGA	AAGGCCCCTGCATAGAAGTT
Cxcr7	Mouse	AGCCTGGCAACTACTCTGACA	GAAGCACGTTCTTGTTAGGCA
Ccr2	Mouse	ATCCACGGCATACTATCAACATC	CAAGGCTCACCATCATCGTAG
Gnai1	Mouse	ACAGACACGTCCATCATCCT	TCTGTTACAGCATCGAACAC
Gnai2	Mouse	GACTTTGCTGATCCCCAG	GTCACTCTGTGCAATGCGC
Gnai3	Mouse	GGGGAGAAAGCGGCCAAA	CTCCCAGCTAAAACAAATAAC

Gnas	Mouse	GGGCTGCCTCGGCAACAG	GCGTGGCCCGGTAGACCTGC
Ch25h	Mouse	TGCTACAACGGTTCGGAGC	AGAAGCCCACGTAAGTGATGAT
Adgre1	Mouse	TTGTACGTGCAACTCAGGACT	GATCCCAGAGTGTTGATGCAA
Mertk	Mouse	CAGGGCCTTTACCAGGGAGA	TGTGTGCTGGATGTGATCTTC
Ifna	Mouse	TCTGATGCAGCAGGTGGG	GGCTCTCCAGACTTCTGCTCTG
Ifna4	Mouse	AAGCCATCCTTGTGCTAAGAGA	AGCAAGTTGGTTGAGGAAGAGA
ACTB	Human	CATGTACGTTGCTATCCAGGC	CTCCTTAATGTCACGCACGAT
EBI2	Human	CACGGCCAGGATAGTAATGCC	CAAGGCTAGTAAGTTTCCCACG
CH25H	Human	ATGTTTGTGTTCCCCGTGAC	GCAGGCAGAACAGGATGTG
CYP7B1	Human	TCTCTTTGCCGCCACCTTAC	AGGCTTTCGCTGATAATCGG
HSD3B7	Human	GGCTGGTAGACGTGTTTGGCAG	TGTAGACCAGGAACCGTGTTCC
LDLR	Human	ACCAACGAATGCTTGGACAAC	ACAGGCACTCGTAGCCGAT
SREBP2	Human	CCTGGGAGACATCGACGAGAT	TGAATGACCGTTGCACTGAAG
SQLE	Human	GGCATTGCCACTTTCACCTAT	GGCCTGAGAGAATATCCGAGAAG
HMGCR	Human	TGATTGACCTTTCCAGAGCAAG	CTAAAATTGCCATTCCACGAGC
DHCR7	Human	GCAGGGGTTGTGAACAAGTAT	GAGACGGCATAGCCAAGGAT
DHCR24	Human	GCCGCTCTCGCTTATCTTCG	GTCTTGCTACCCTGCTCCTT
CYP51A1	Human	GAAACGCAGACAGTCTCAAGA	ACGCCCATCCTTGTATGTAGC
ABCA1	Human	ACATCCTGAAGCCAATCCTGA	CTCCTGTCGCATGTCACTCC
ABCG1	Human	GGGGTCGCTCCATCATTTG	TTCCCCGGTACACACATTGTC
ACAT1	Human	ATGCCAGTACACTGAATGATGG	GATGCAGCATATACAGGAGCAA
ACAT2	Human	GCGGACCATCATAGGTTCCTT	ACTGGCTTGTCTAACAGGATTCT
CYP27A1	Human	GGTGCTTTACAAGGCCAAGTA	TCCCGGTGCTCCTTCCATAG
CYP46A1	Human	TGTGTTTTTGGATTGGGCTAAGA	ACTCAGGACTCGTGACGATGA
CYP7A1	Human	GCAATTTGGTGCCAATCCTCT	GCACAACACCTTATGGTATGACA
CYP39A1	Human	CTTCAGCGGAAGAATTTGCGT	AAAGGTCATTCGGTTTCCCATAG
NPC1L1	Human	AGAGTGAGCCTTACACAACCA	GCAGGACACGTTGGAGAGT
MYLIP	Human	GCAGGCGACTGGGAATCATAG	CGGTTTCTCAGGTTTAGCCAT
LIMA1	Human	GACTCCCAGGTTAAGAGTGAGG	TTGCAGGTGCCTGAAACTTCT

HMGCS1	Human	CTCTTGGGATGGACGGTATGC	GCTCCAACTCCACCTGTAGG
FDFT1	Human	CCACCCCGAAGAGTTCTACAA	TGCGACTGGTCTGATTGAGATA
CYP11A1	Human	GCAGTGTCTCGGGACTTCG	GGCAAAGCGGAACAGGTCA
CYP17A1	Human	GCTGCTTACCCTAGCTTATTTGT	ACCGAATAGATGGGGCCATATTT
CYP11B1	Human	GGGTGGCCTACAGACAACATC	GGCGACAGCACTTCTGGATT
HSD11B1	Human	TGGCTTATCATCTGGCGAAGA	AGGCAGTGGGATACCACCT
HSD11B2	Human	CCACCGTATTGGAGTTGAACA	CGCGGCTAATGTCTCCTGG
IFNB1	Human	GCTTGGATTCCTACAAAGAAGCA	ATAGATGGTCAATGCGGCGTC
IFNA	Human	GCTTGGGATGAGACCCTCCTA	CCCACCCCCTGTATCACAC

Supplementary Figures and Figure legends

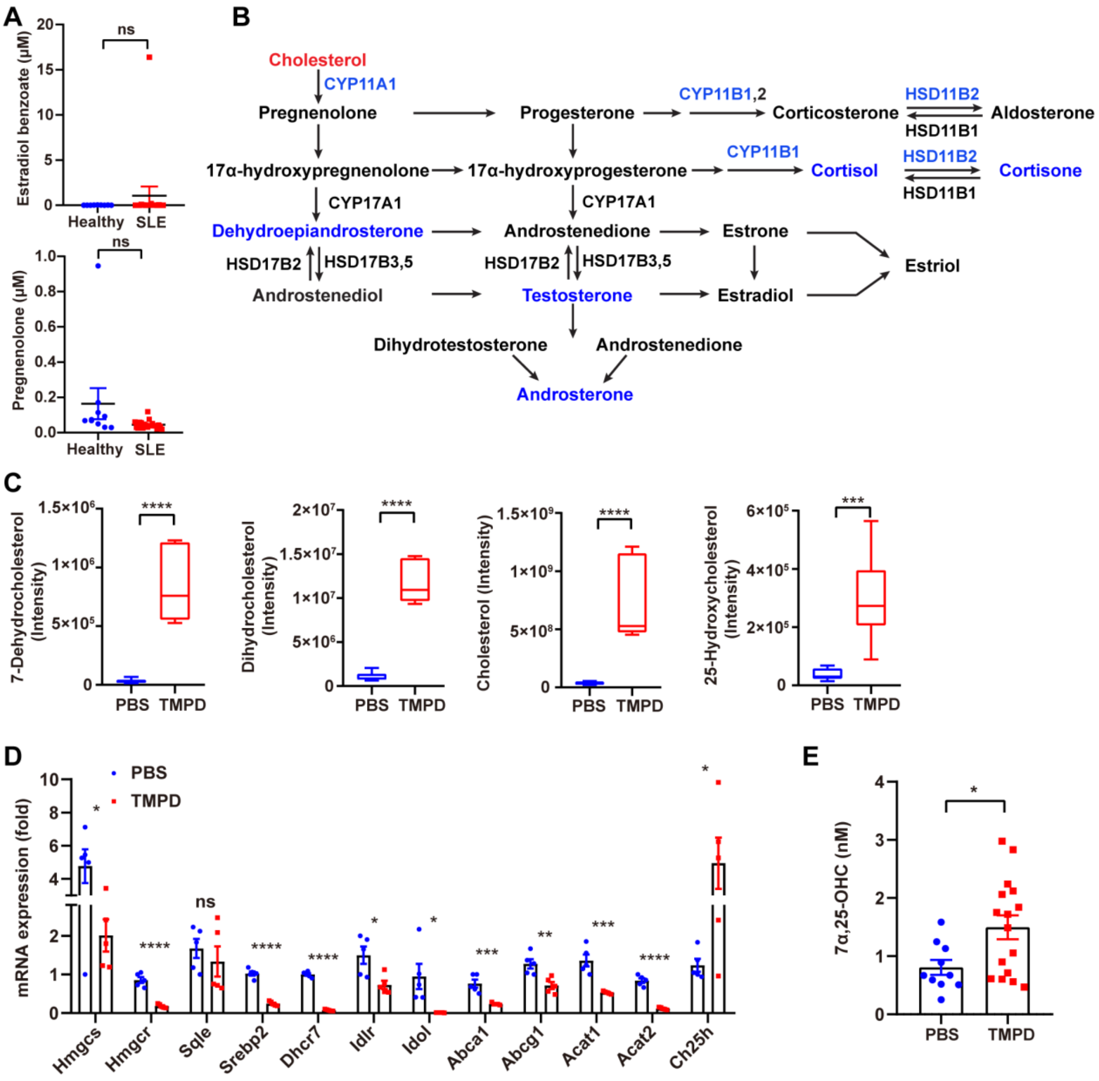

Figure S1. Abnormal cholesterol metabolism in SLE patients and mice.

A) Plasma concentrations of estradiol benzoate and pregnenolone in HCs (n= 10) and SLE patients (n = 20) by LC-MS. B) Schematic diagram of the steroid hormones biosynthesis pathway. The up-regulated intermediates or enzyme genes in SLE patients were shown in red, while the down-regulated intermediates or enzyme genes were shown in blue. C) The intensity of 7-DHC, dihydrocholesterol, cholesterol, and 25-HC in PIF from PBS- and TMPD-treated mice by LC-MS (n=8). D) The relative mRNA expression of cholesterol metabolism-related genes in peritoneal cells of PBS- and TMPD- treated mice (n= 5). E）The concentration of 7α, 25-OHC in the serum of TMPD-treated mice (n = 16) and PBS controls (n = 10) by LC-MS. Data are shown as

mean ± SEM. *P < 0.05, **P < 0.01, ***P < 0.001, and ****P < 0.0001, using a two-tailed, unpaired Student's t test (A, C, and E), or two-way ANOVA with Holm-Sidak's multiple comparisons test (D).

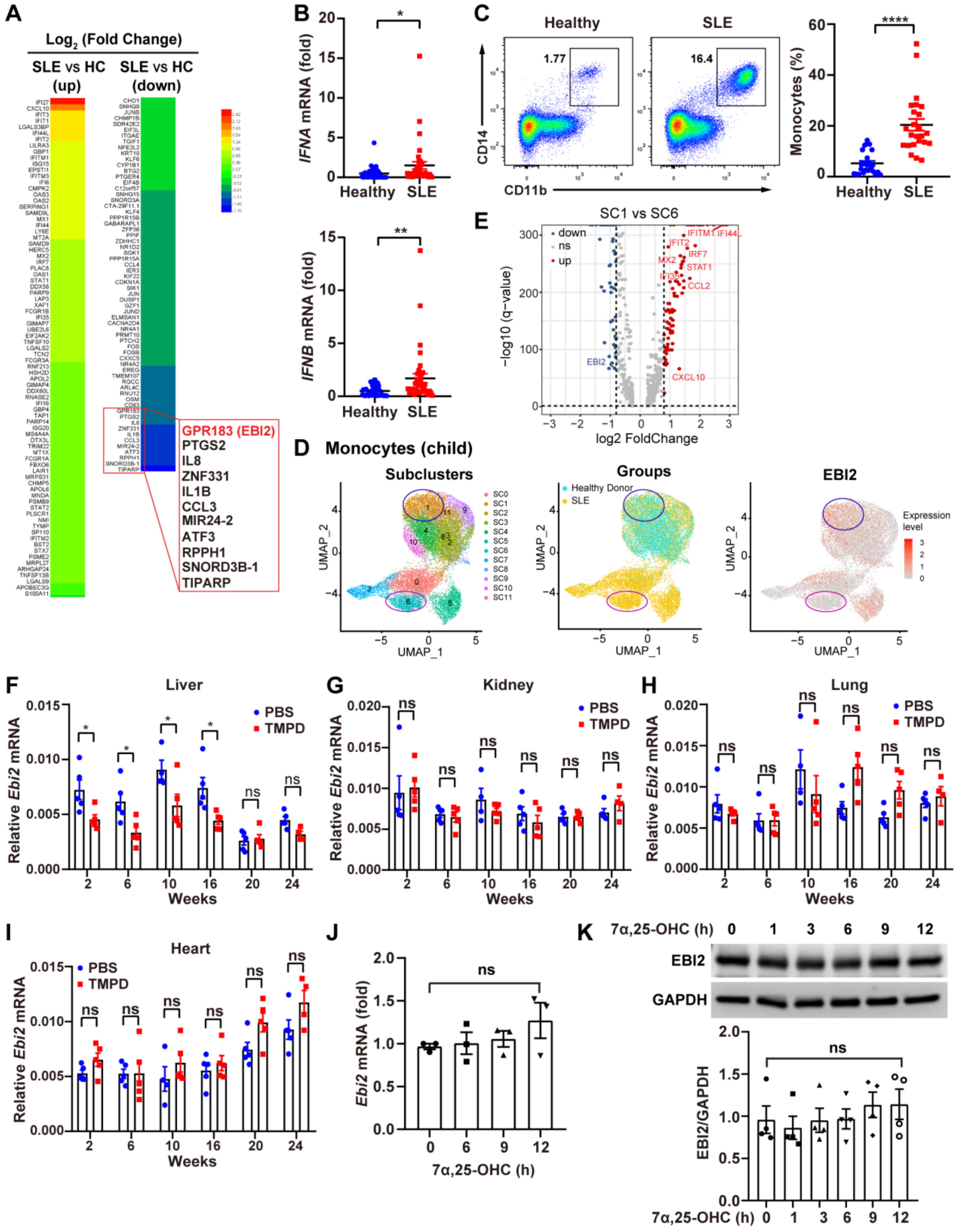

Figure S2. The expression of EBI2 in monocytes of SLE patients or tissues of SLE mice.

A) Heat map of DEGs in cMos of SLE patients with Asian ancestry versus those in HCs. B) The mRNA expression of *IFNA* and *IFNB* in PBMCs of healthy donors (n = 40) and SLE patients (n = 36). C) Flow cytometric analysis of monocytes in PBMCs of healthy donors (n = 24) and SLE patients (n = 24). D) scRNA-seq data of

monocytes from pediatric SLE patients or healthy donors were classified into molecularly distinct subclusters. UMAP plots representing SCs (n = 11), groups (Healthy donors or SLE), and the expression values of EBI2. (E) Volcano plot of DEGs from SC1 (EBI2^{hi}) and SC6 (EBI2^{lo}). F-I) mRNA levels of *Ebi2* in liver, kidney, lung and heart of mice with PBS (n = 5, and n = 4 only at week 10) or TMPD (n = 5, and n = 4 only at week 24) treatment for different time. J, K) WT PEMs were treated with 10 μM 7α, 25-OHC for different time to detect the mRNA (n = 3) (J) or protein (n = 4) levels of EBI2 (K). Data are shown as mean ± SEM. *P < 0.05, **P < 0.01 and ****P < 0.0001, using a two-tailed, unpaired Student's t test (B, C right panel), two-way ANOVA with Holm-Sidak's multiple comparisons test (F-I), or one-way ANOVA with Holm-Sidak's multiple comparisons test (J and K).

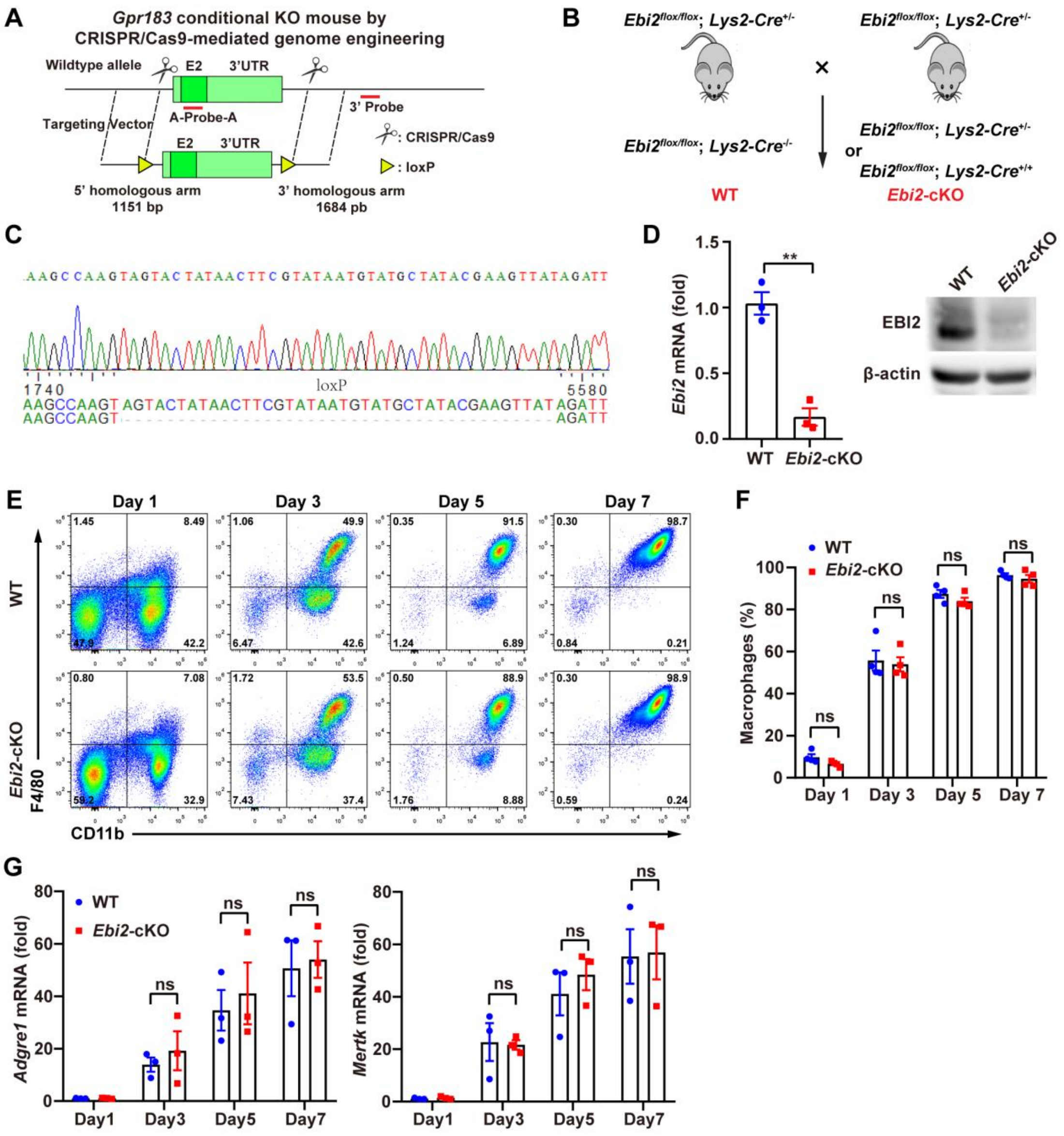

Figure S3. ***Ebi2*****-cKO mice show normal macrophages development.**

A) The exon 2 in the *Ebi2* gene was floxed to generate *Ebi2* conditional knockout mice by CRISPR/Cas9 strategy. B-D) $Ebi2^{fl/fl}$ mice were crossbred with *Lyz2-Cre* mice to delete EBI2 in myeloid cells (B). *Ebi2* deletion was confirmed by genome sequencing (C), or in PEMs by qRT-PCR and immunoblot (D, n= 3). E-G) WT and *Ebi2*-cKO BMs were induced by M-CSF (20 ng/ml) for different days to detect the percentages of BMDMs in WT and *Ebi2*-cKO mice (E and F, n= 4), or to measure the mRNA levels of *Adgre1* and *Mertk* (G, n= 3). Data are shown as mean ± SEM. $^{**}P < 0.01$, using a two-tailed, unpaired Student's *t* test (D), or two-way ANOVA with Holm-Sidak's multiple comparisons test (F and G).

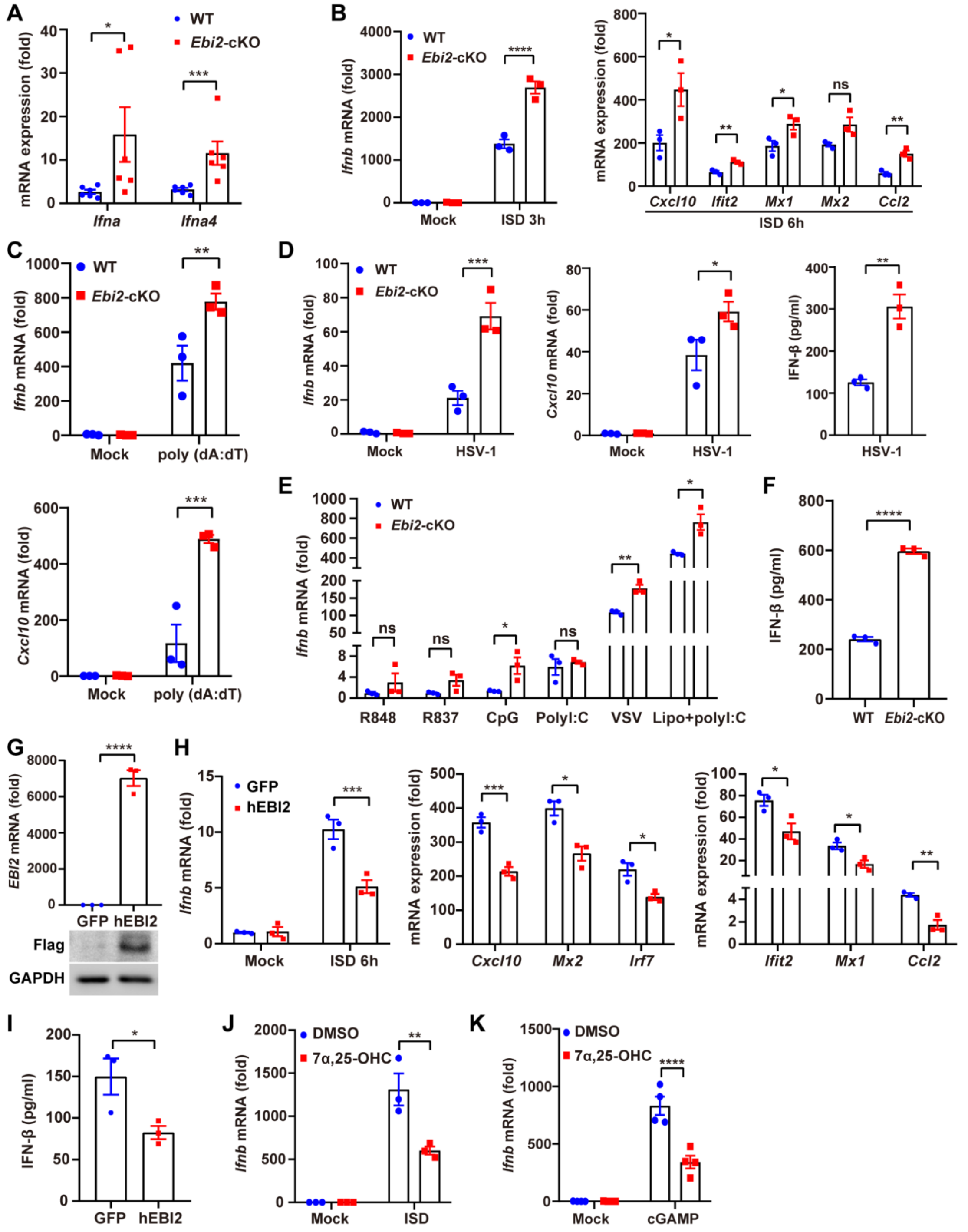

Figure S4. EBI2 suppresses IFN-β production.

A) WT (n = 6) and *Ebi2*-cKO (n = 6) PEMs were treated with QVD-OPh and ABT-737 for 24 h to detect the mRNA of *Ifna* and *Ifna4*. B-D) WT (n = 3) and *Ebi2*-cKO (n = 3) PEMs were stimulated with ISD, poly (dA:dT) (0.5 μg/ml), or infected with HSV-1 (MOI, 1) to detect the transcription of *Ifnb* and ISGs. (E, F) WT (n = 3) and *Ebi2*-cKO (n = 3) PEMs were stimulated with dual TLR7 and TLR8

agonist Resiquimod (R848), TLR7 agonist lmiquimod (R837), TLR9 agonist CpG, TLR3 and RIG-I agonist poly (I:C) or infected with VSV to detect the transcription of *Ifnb* (E), or production of IFN-β in culture supernatant of poly (I:C)-transfected cells (F). G) iBMDMs were stably transfected with human EBI2 (hEBI2) using retrovirus to detect EBI2 expression by qRT-PCR and immunoblot (n = 3). H, I) iBMDM-GFP/hEBI2 were stimulated with ISD for 6 h to detect the mRNA levels of *Ifnb, Cxcl10, Mx1, Mx2, Irf7, Ifit2* and *Ccl2* (n = 3) and protein level of IFN-β (n = 3). J) PEMs were pre-treated with 7α, 25-OHC followed by ISD stimulation to detect the transcription of *Ifnb* (n = 3). K) PEMs were pre-treated with cGAMP (0.1μg/ml, 30 min) followed by 7α, 25-OHC treatment to detect the transcription of *Ifnb* (n = 4). Data are shown as mean ± SEM. $*P < 0.05$, $**P < 0.01$, $***P < 0.001$, and $****P < 0.0001$, using two-way ANOVA with Holm-Sidak's multiple comparisons test (B left panel, C, D, H left panel, J and K), or a two-tailed, unpaired Student's *t* test (A, B right panel, F, G up panel, H right panel, I).

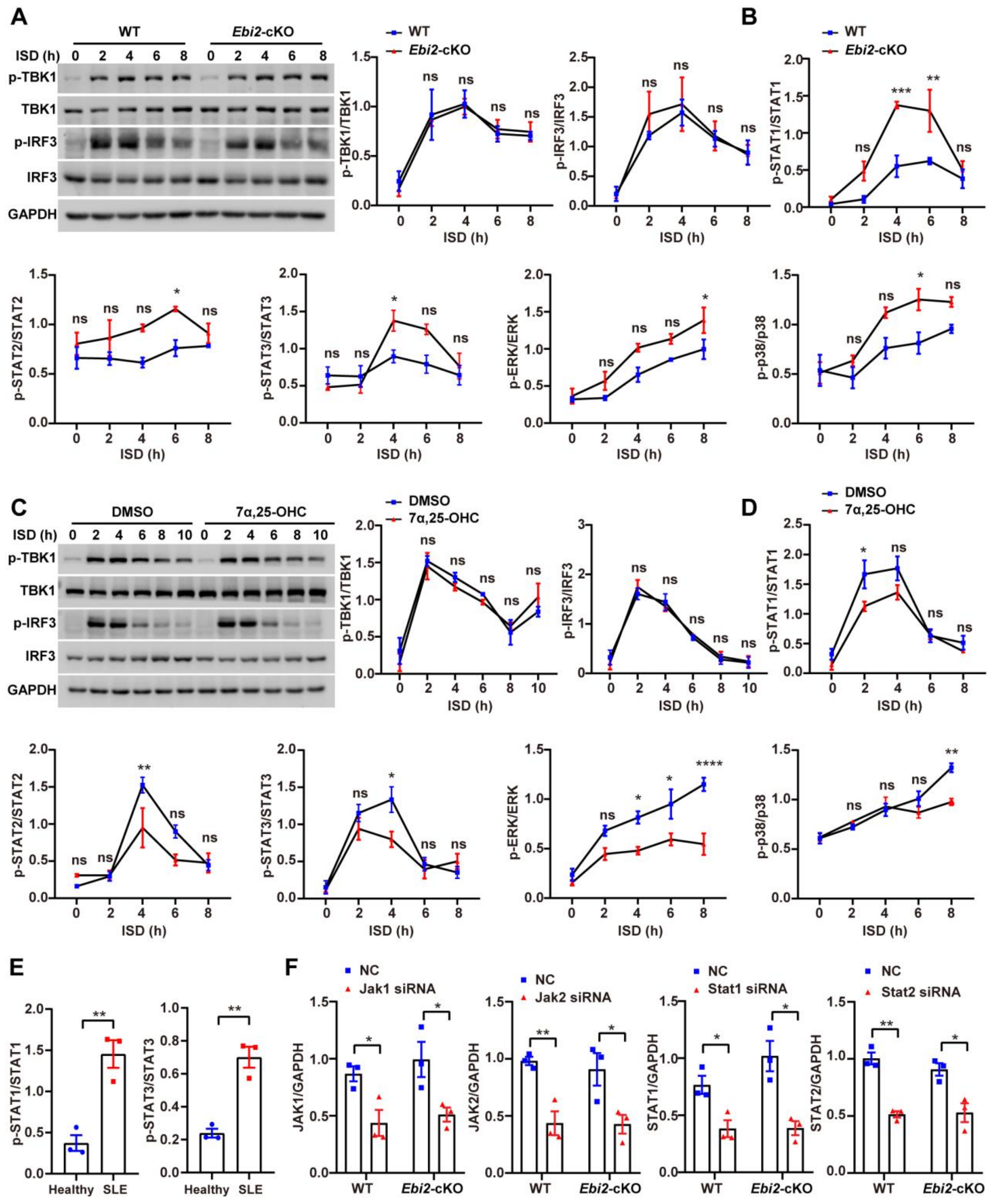

Figure S5. EBI2 does not affect the phosphorylation of TBK1 and IRF3.

A) WT and *Ebi2*-cKO PEMs were stimulated with ISD to detect and quantify the phosphorylation levels of TBK1 and IRF3 (n = 3). B) WT and *Ebi2*-cKO PEMs were stimulated with ISD to quantify the phosphorylation levels of STAT1, STAT2, STAT3, ERK and p38 (n = 3). C) PEMs were pre-treated with 7α, 25-OHC followed by ISD stimulation to detect and quantify the phosphorylation levels of TBK1 and IRF3 (n = 3). D) PEMs were pre-treated with 7α, 25-OHC followed by ISD stimulation to

quantify the phosphorylation levels of STAT1, STAT2, STAT3, ERK and p38 (n = 3). E) The phosphorylation levels of STAT1 and STAT3 were quantified in PBMCs of SLE patients (n = 3) and HCs (n = 3). F) WT and *Ebi2*-cKO PEMs were transfected with siRNAs targeting *Jak1*, *Jak2*, *Stat1*, and *Stat2* for 48 h to quantify the protein levels of JAK1, JAK2, STAT1 and STAT2 (n = 3). Data are shown as mean ± SEM. *P < 0.05, **P < 0.01, ***P < 0.001, and ****P < 0.0001, using two-way ANOVA with Holm-Sidak's multiple comparisons test (A-D, and F), or a two-tailed, unpaired Student's *t* test (E).

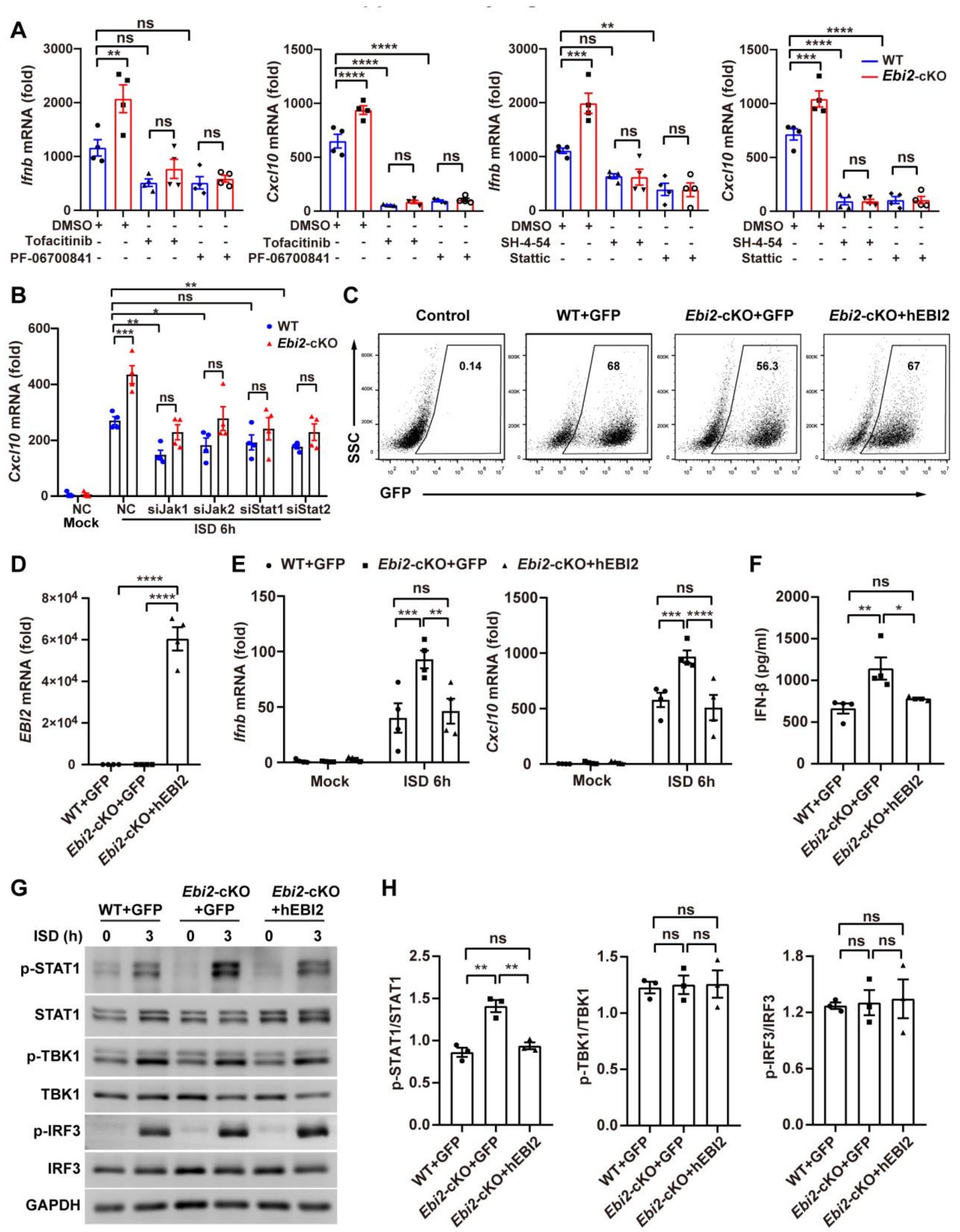

Figure S6. Replenishment of EBI2 in *Ebi2*-cKO macrophages inhibits IFN-I response.

A) PEMs were pre-treated with Tofacitinib (10 μM), PF-06700841 (10 μM), SH-4-54 (10 μM), or Stattic (10 μM) for 2 h followed by ISD stimulation for 3 h to measure the transcription of *Ifnb* and *Cxcl10* (n = 4). B) PEMs were transfected with siRNAs

targeting *Jak1*, *Jak2*, *Stat1*, and *Stat2* for 48 h followed by ISD stimulation to measure the transcription of *Cxcl10* (n = 4). C, D) Primary *Ebi2*-cKO BMDMs were stably transfected with hEBI2 to detect the percentage of GFP^+ cells (C) and the mRNA level of *EBI2* (D, n = 4). E, F) GFP or hEBI2 overexpressed WT and *Ebi2*-cKO BMDMs were stimulated with ISD for 6 h to detect the mRNA levels of *Ifnb* and *Cxcl10* (n = 4) (E) or protein level of IFN-β (n = 4) (F). G, H) GFP or hEBI2 overexpressed WT and *Ebi2*-cKO BMDMs were stimulated with ISD for 3h to detect and quantify the phosphorylation levels of STAT1, TBK1 and IRF3 (n = 3). Data are from three independent experiments (mean ± SEM). $*P < 0.05$, $**P < 0.01$, $***P < 0.001$, and $****P < 0.0001$, using two-way ANOVA with Holm-Sidak's multiple comparisons test (A, B and E), or one-way ANOVA with Holm-Sidak's multiple comparisons test (D, F and H).

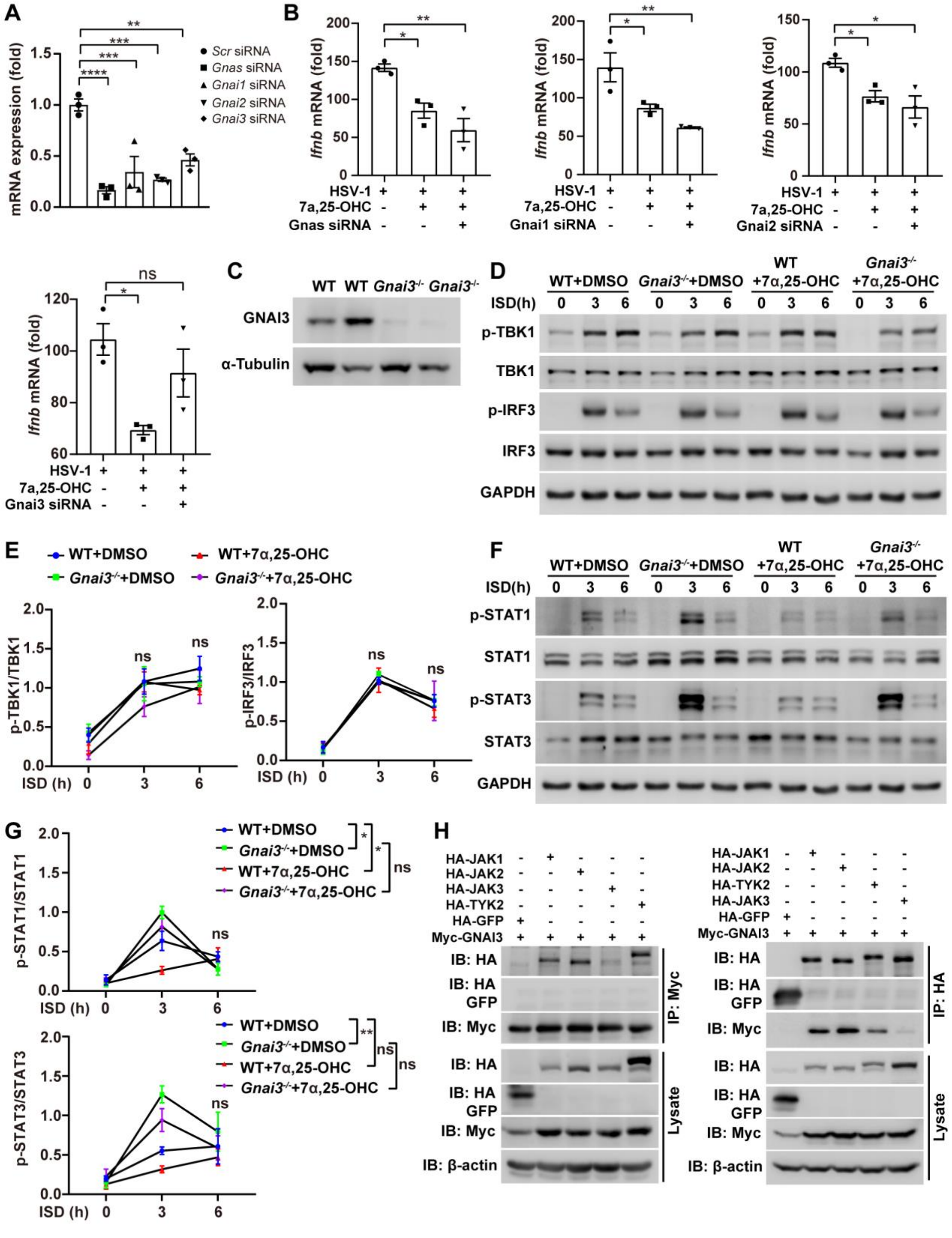

Figure S7. EBI2 inhibits IFN-I response through GNAI3.

A, B) PEMs were transfected with siRNAs (40 nM) to respectively silence *Gnas*, *Gnai1*, *Gnai2*, and *Gnai3* for 48 h, and then pre-treated with 7α, 25-OHC for 2 h followed by HSV-1 (MOI, 1) infection for 6 h to detect the transcription of *Ifnb* (n = 3). C) The *Gnai3* KO efficiency in BMDMs was confirmed by immunoblot. D-G) WT

and Gnai3$^{-/-}$ BMDMs were treated with DMSO or 7α, 25-OHC (10 μM) for 2 h followed by ISD stimulation for 3 or 6 h to detect and quantify the phosphorylation levels of TBK1, IRF3, STAT1, STAT3 (n= 3). H) HA-tagged JAK1, JAK2, JAK3, and TYK2 were transfected with Myc-tagged GNAI3 to detect their interaction by immunoprecipitation and immunoblot. Data are shown as mean ± SEM. *P < 0.05, **P < 0.01, ***P < 0.001 and ****P < 0.0001, using one-way ANOVA with Holm-Sidak's multiple comparisons test (A and B), or using two-way ANOVA with Holm-Sidak's multiple comparisons test (E and G).

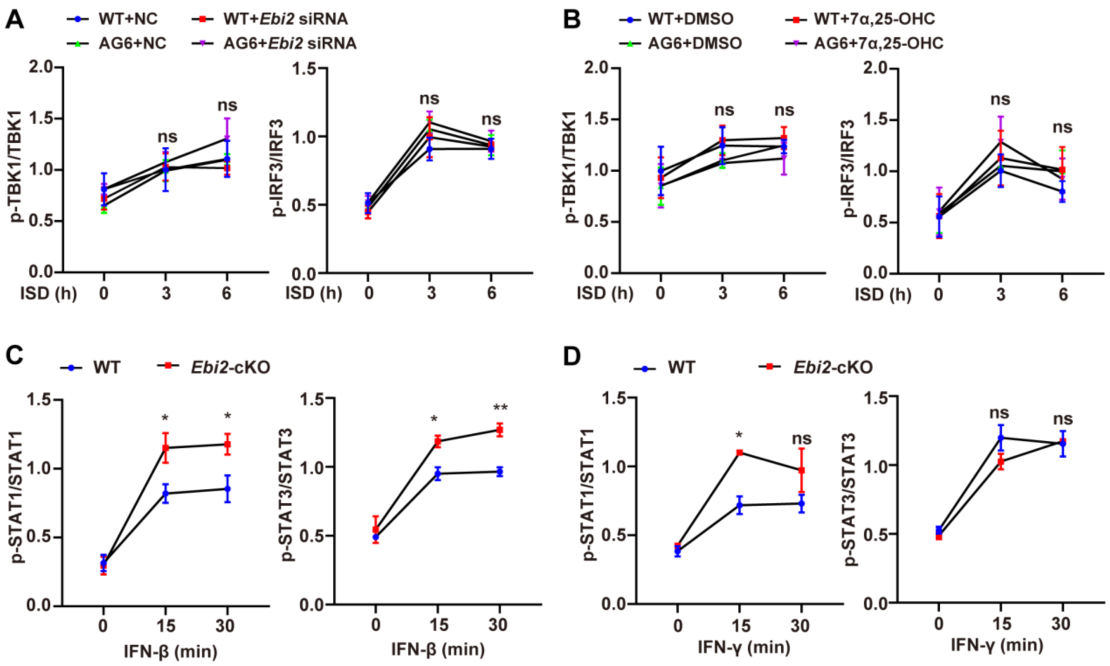

Figure S8. Quantitative analysis of protein phosphorylation.

A, B) WT and AG6 PEMs were transfected with *Ebi2* siRNA for 48 h (A, n = 3), or treated with DMSO or 7α, 25-OHC for 2 h (B, n = 3), followed by ISD stimulation for 3 or 6 h to quantify the phosphorylation levels of TBK1 and IRF3. C, D) WT and *Ebi2*-cKO PEMs were stimulated with IFN-β (500 ng/ml) or IFN-γ (200 ng/ml) for 15 or 30 min to quantify the phosphorylation levels of STAT1 and STAT3 (n = 3). Data are shown as mean ± SEM. **P* < 0.05, ***P* < 0.01, using two-way ANOVA with Holm-Sidak's multiple comparisons test (A-D).

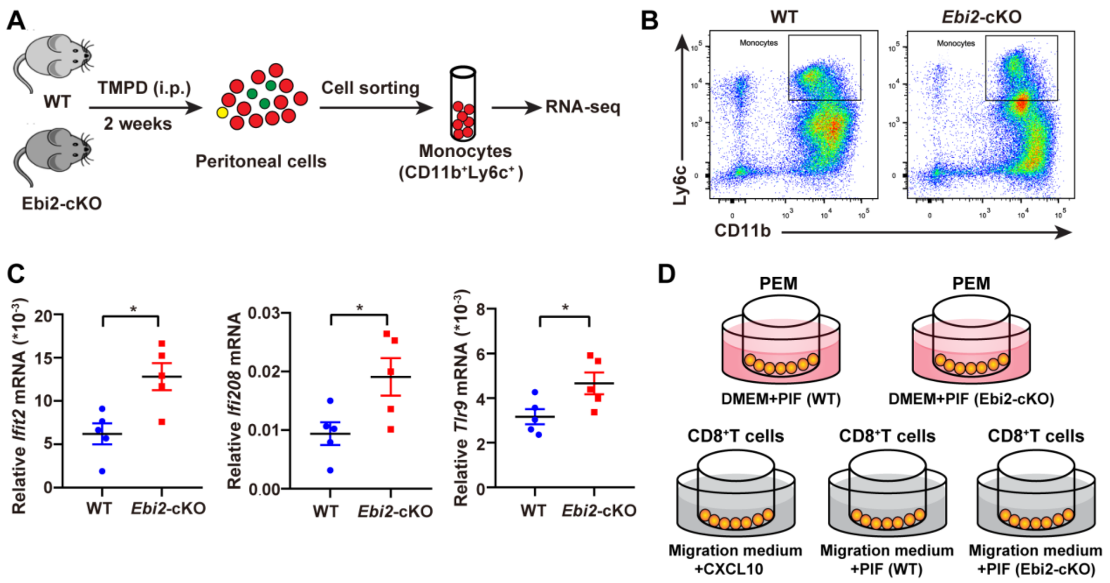

Figure S9. Analysis of peritoneal monocytes in TMPD-induced mice.

A, B) Flow charts showing how to sort monocytes from peritoneal cells of TMPD-treated WT and *Ebi2*-cKO mice for RNA-seq. C) The expression of *Ifit2*, *Ifi208*, and *Tlr9* in peritoneal monocytes of TMPD-treated WT (n = 5) and *Ebi2*-cKO mice (n = 5). D) Design of the transwell experiments. Data are shown as mean ± SEM. **P* < 0.05 using a two-tailed, unpaired Student's *t* test (C).

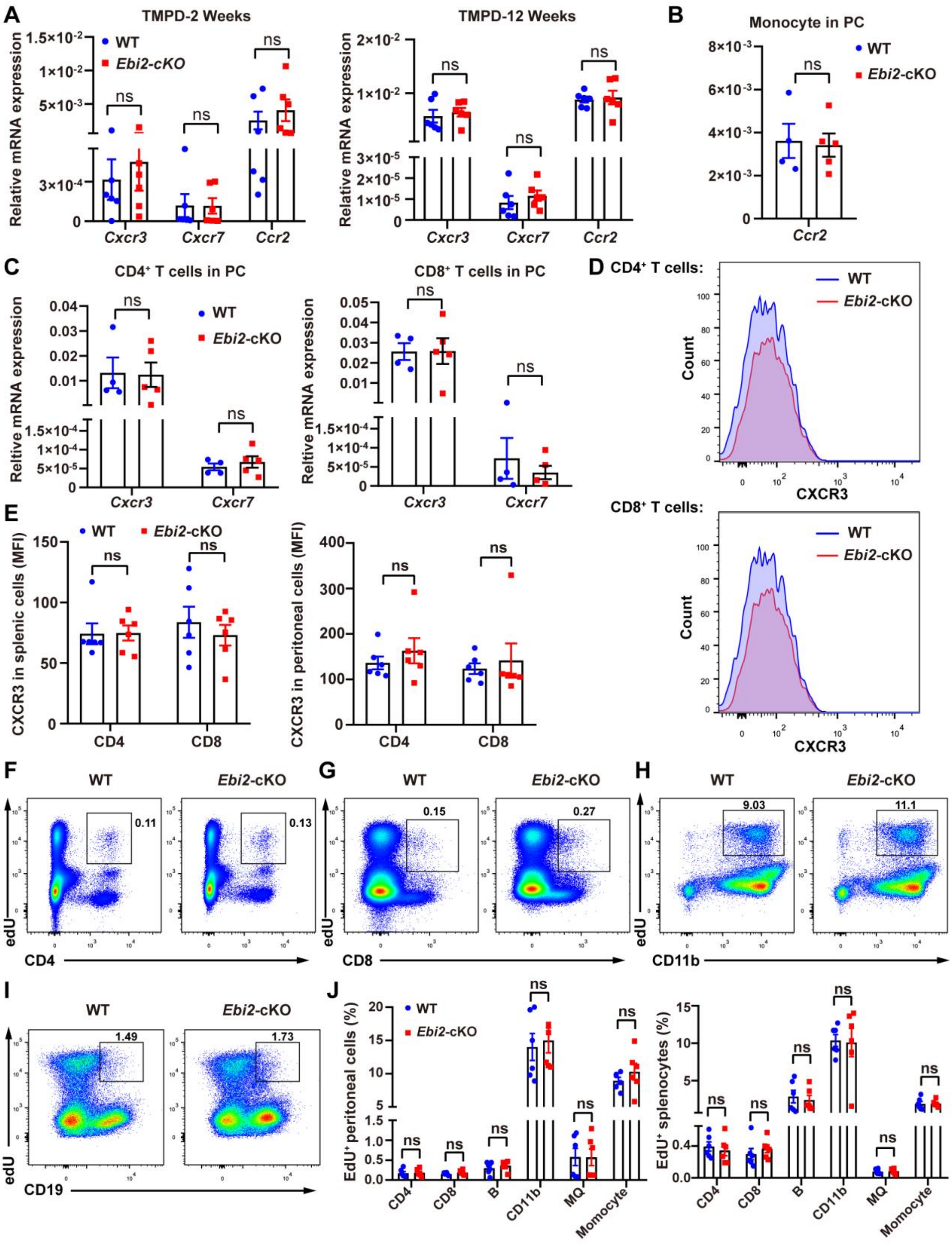

Figure S10. EBI2 deficiency in macrophages does not affect CXCR3/CCR2 expression or cell proliferation.

A) The mRNA expression of *Cxcr3*, *Cxcr7* and *Ccr2* in peritoneal cells of TMPD-treated WT (n = 6) and *Ebi2*-cKO mice (n = 6). (B, C) The mRNA expression of *Ccr2* in peritoneal monocytes (B), or *Cxcr3*, *Cxcr7* in peritoneal CD4^{+} and CD8^{+} T

cells (C) of TMPD-treated WT (n = 4) and *Ebi2*-cKO (n = 5) mice. D, E) Representative histograms and quantitative analyses of CXCR3 in splenic or peritoneal $CD4^+$ and $CD8^+$ T cells from WT (n = 6) and *Ebi2*-cKO (n = 6) mice after TMPD treatment for 12 wk. F) Representative dot plots of EdU staining in peritoneal $CD4^+$ T, $CD8^+$ T, and $CD11b^+$ cells (F-H) or in splenic B cells (I) from WT and *Ebi2*-cKO mice after TMPD treatment for 12 wk. J) Statistical analysis of the percentages of EdU^+ immune cells in TMPD-treated WT (n = 6) and *Ebi2*-cKO (n = 6) mice. Data are shown as mean ± SEM. Ns, not significant (P > 0.05), using two-way ANOVA with Holm-Sidak's multiple comparisons test (A, C, E and J), or using a two-tailed, unpaired Student's *t* test (B).

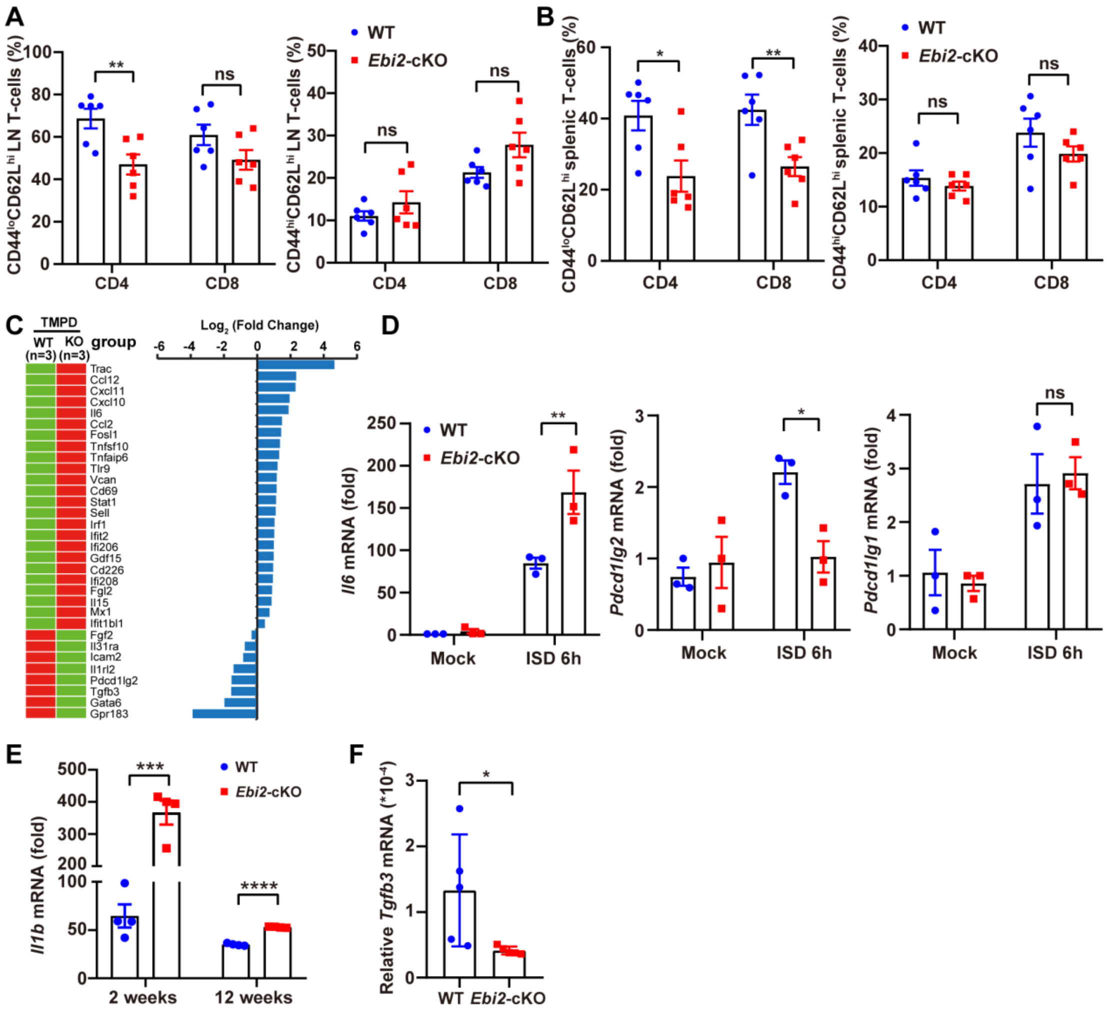

Figure S11. EBI2 deficiency in macrophages promotes lymphocyte activation.

A, B) Percentages of naïve (CD44^{lo} CD62L^{hi}) and central memory (CD44^{hi} CD62L^{hi}) T cells in LNs or spleens of WT (n = 6) or *Ebi2*-cKO (n = 6) mice after TMPD treatment for 12 wk. C) Partial DEGs in peritoneal monocytes of TMPD-induced WT (n = 3) or *Ebi2*-cKO (n = 3) mice were listed. D) WT (n = 3) or *Ebi2*-cKO PEMs (n = 3) was stimulated with ISD for 6 h to detect the transcription of *Il6*, *Pdcd1lg1*, and *Pdcd1lg2*. E) *Il1b* mRNA expression was detected in peritoneal cells of WT (n = 4) or *Ebi2*-cKO (n = 4) mice after TMPD treatment for 2 or 12 wk. F) mRNA expression of *Tgfb3* was examined in peritoneal monocytes of WT (n = 5) and *Ebi2*-cKO (n = 5)mice after TMPD treatment for 2 wk. Data are shown as mean ± SEM. **P* < 0.05, ***P* < 0.01 and ****P* < 0.001, using two-way ANOVA with Holm-Sidak's multiple comparisons test (A, B, D and E), or a two-tailed, unpaired Student's *t* test (F).
